# Cranial shape evolution in adaptive radiations of birds: comparative morphometrics of Darwin's finches and Hawaiian honeycreepers

**DOI:** 10.1098/rstb.2015.0481

**Published:** 2017-02-05

**Authors:** Masayoshi Tokita, Wataru Yano, Helen F. James, Arhat Abzhanov

**Affiliations:** 1 Department of Organismic and Evolutionary Biology, Harvard University, Cambridge, MA 02138, USA; 2 Department of Oral Anatomy, Asahi University School of Dentistry, 1851 Hozumi, Mizuho, Gifu 501-0296, Japan; 3 Department of Vertebrate Zoology, National Museum of Natural History, Smithsonian Institution, MRC 116, Washington, DC 20013-7012, USA

**Keywords:** adaptive radiation, geometric morphometrics, Darwin's finches, Hawaiian honeycreepers, skull shape

## Abstract

Adaptive radiation is the rapid evolution of morphologically and ecologically diverse species from a single ancestor. The two classic examples of adaptive radiation are Darwin's finches and the Hawaiian honeycreepers, which evolved remarkable levels of adaptive cranial morphological variation. To gain new insights into the nature of their diversification, we performed comparative three-dimensional geometric morphometric analyses based on X-ray microcomputed tomography (µCT) scanning of dried cranial skeletons. We show that cranial shapes in both Hawaiian honeycreepers and Coerebinae (Darwin's finches and their close relatives) are much more diverse than in their respective outgroups, but Hawaiian honeycreepers as a group display the highest diversity and disparity of all other bird groups studied. We also report a significant contribution of allometry to skull shape variation, and distinct patterns of evolutionary change in skull morphology in the two lineages of songbirds that underwent adaptive radiation on oceanic islands. These findings help to better understand the nature of adaptive radiations in general and provide a foundation for future investigations on the developmental and molecular mechanisms underlying diversification of these morphologically distinguished groups of birds.

This article is part of the themed issue ‘Evo-devo in the genomics era, and the origins of morphological diversity’.

## 1. Introduction

**Figure 1.**
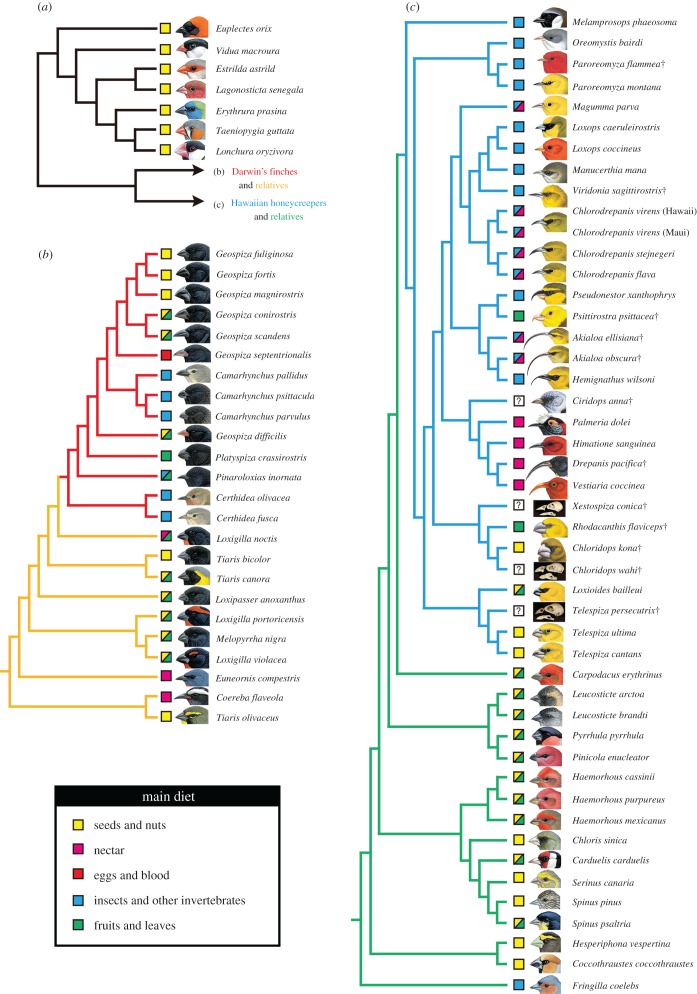
The phylogeny of bird species used for mapping shape data by squared-change parsimony. (*a*) The phylogeny of the representative species of Ploceidae and Estrildidae that are used as outgroups. The topology was based on [9] where the phylogenetic relationship between congener species was inferred. (*b*) The phylogeny of Darwin's finches (red branches) and their relatives (subfamily Coerebinae, family Thraupidae; orange branches). The relationship among basal species was based on molecular phylogeny in [10]. Basic structure of the phylogeny of Darwin's finches was after molecular phylogeny in [8], except for the phylogeny among three ground and two cactus finch species that was derived from the phylogeny in [11]. (*c*) The phylogeny of Hawaiian honeycreepers (often classified as subfamily Drepanidinae; blue branches) and their relatives (other species in the family Fringillidae; green branches). The basic structure of the phylogeny follows the molecular phylogeny in [12]. The phylogenetic position of each extinct species (indicated by †) was determined by considering its position in the osteology-based phylogeny in [13]. Information about the main diet of each species was taken from [14], except for diet information about extinct Hawaiian honeycreepers, which was derived from [15]. Diet of the species that eat two types of food belonging to distinct categories approximately equally is shown with two colours. Illustrations of the head of each bird species were derived from [14], except for those of extinct Hawaiian honeycreeper species that were derived from [15] with the author's permission. Pictures of the skulls of three extinct Hawaiian honeycreeper species were taken by Caitlin Price. Illustrations of Hawaiian honeycreepers are by H. Douglas Pratt; illustrations of all other birds are by Elisa Badia. Taxonomic nomenclature of each bird species is after the IOC World Bird List [16].

Adaptive radiation, or the rapid evolution of morphologically and ecologically diverse species from a single ancestor [1,2], was first described as an important phenomenon in organismal evolution in *The major features of evolution* by George Gaylord Simpson [3]. Two classic and the most striking examples of adaptive radiation in animals are found among the songbirds: Darwin's finches and the Hawaiian honeycreepers. Darwin's finches are a member of the tanagers (the family Thraupidae) [4] and endemic to the Galápagos Islands and Cocos Island [5–7]. They comprise 16 living species [8] (figure 1), and no species are known to have become extinct after human colonization of the Galápagos archipelago. Darwin's finches belong to the subfamily Coerebinae (the ‘dome-nest’ tanagers), a group that includes 14 additional species, such as grassquits, bullfinches and the bananaquit, all found in Central and South America and the Caribbean islands [4,10]. Thus, Coerebinae started as a West Indian radiation that colonized the mainland and eventually Galápagos [4]. Molecular phylogenies [4,17] indicate that the ancestral species of Darwin's finches likely colonized the Galápagos Islands between 4 and 2.5 Ma. The Hawaiian honeycreepers are representatives of the true finches (Fringillidae) that are endemic to the Hawaiian Islands [15] and comprise 18 extant species (including the recently extinct poo-uli) and over 30 extinct species [13,18] (figure 1). A recent molecular phylogenetic study proposed that they are closely related to Eurasian rosefinches (*Carpodacus*) (figure 1) and the ancestral colonists arrived in Hawaii from Eastern Asia around 8–6 Myr with the earliest divergence within the honeycreeper clade dated to around 6–5 Ma [12].

One of the major characteristics of adaptive radiation in animals is the morphological diversification that is functionally related to the utilization of different types of resources following the expansion into a variety of unoccupied ecological niches [19–24]. The avian bill (upper and lower beaks together) is the key force-transmitting structure used for feeding in birds [25–28], and its shape can vary dramatically during adaptive radiation. Such remarkable diversity of beak morphology both in terms of size and shape has been previously noted and quantitatively described in both Darwin's finches [29–32] and Hawaiian honeycreepers [33,34] with a focus on the upper beak. However, not much is known about morphological diversity in the cranium portion of the bird skull (cranial vault and neurocranium), where the jaw muscles originate and whose shape influences the mechanics of the jaw muscles [26,35–39], in the context of avian adaptive radiation.

**Figure 2.**
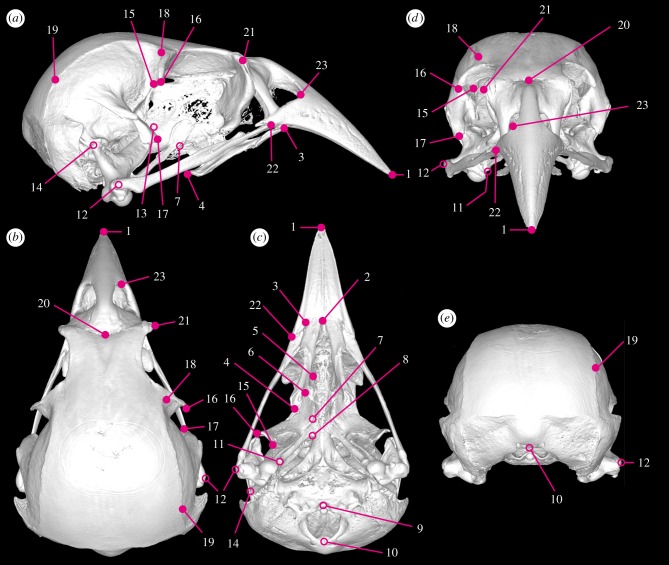
Landmarks digitized on the skull. (*a*) Lateral, (*b*) dorsal, (*c*) ventral, (*d*) frontal and (*e*) caudal views of the skull. When the skull specimens were damaged or broken, the landmarks 7, 8, 9, 10, 11, 12, 13 and 14 (indicated by open circles) were excluded from the PCA. The three-dimensional µCT image shown here is the skull of a large cactus finch (*Geospiza conirostris*; CAS86666). Definitions of the landmarks are given in the electronic supplementary material, table S2.

The most important goal of this study is to gain new insights into the magnitude and modes of morphological diversification of the skulls in avian adaptive radiations on oceanic islands by quantitatively analysing and comparing the shapes of skulls in both Darwin's finches and Hawaiian honeycreepers (including some extinct/fossil taxa), their respective relatives and shared outgroup taxa (figure 1; electronic supplementary material, table S1). We employed landmark-based three-dimensional geometric morphometric (GMM) analysis (figure 2; electronic supplementary material, table S2), which enables the quantification of morphological variation in complex three-dimensional structures, such as vertebrate skulls [40–44], using X-ray microcomputed tomography (µCT) scanning of museum-preserved dried cranial skeletons.

First, we analysed skull morphological disparity using principal component analysis (PCA) [45] to uncover major patterns of variation. By projecting phylogeny into a multivariate morphospace (phylomorphospace *sensu* Sidlauskas [46]), we map the history of each group's morphological diversification to investigate the evolutionary order of morphological transformations, the degree of morphological convergence and the partitioning of morphological diversity among the subclades. Second, we examine which part of the skull predominantly contributes to morphological diversification in each songbird group by quantitatively comparing morphology of several functional subdivisions within the skull (i.e. upper beak, orbit, palatine, pterygoid, quadrate and adductor chamber) using PCA. Third, we evaluate how the whole-skull shape covaries with the shapes of each cranial subdivision in Darwin's finches, Hawaiian honeycreepers and all remaining taxa using the two-block partial least-squares (PLS) analysis [45], to address whether the mode of skull shape evolution through adaptive radiation, which is characterized by rapid morphological diversification, is in large part shared or divergent in the two songbird lineages and whether it matches the trends reported for other case studies on avian morphological evolution [40,47,48]. Finally, using PCA we describe the evolutionary pattern of shape change for the whole skull and each of the skull subdivisions within both Darwin's finches and Hawaiian honeycreepers, and attempt to find similarities and differences in morphological evolution of the skulls between two clades.

## 2. Material and methods

### (a) Specimens

We sampled a total of 101 individuals of 14 species of Darwin's finches; 50 individuals of 10 species in the subfamily Coerebinae, close relatives of Darwin's finches; 100 individuals of 30 species of Hawaiian honeycreepers, including extinct/fossil species; 68 individuals of 16 species in the family Fringillidae, close relatives of the Hawaiian honeycreepers. We chose the families Estrildidae and Ploceidae as the outgroup to all other songbird species studied, considering current molecular phylogenetic frameworks where they are closely related to the clade containing Coerebinae and Fringillidae, and sampled 34 individuals out of seven taxa. Dry cranial skeletons of birds were borrowed from natural history museums in the USA: The American Museum of Natural History (AMNH), The Bernice Pauahi Bishop Museum (BBM/BPBM), California Academy of Sciences (CAS), The Museum of Comparative Zoology, Harvard University (MCZ), The Museum of Vertebrate Zoology, University of California at Berkeley (MVZ) and National Museum of Natural History, Smithsonian Institution (NMNH/USNM). Mostly, male bird skeletons were used for the analyses. However, both male and female skeletons were analysed for Hawaiian honeycreepers and their close relatives because of the rarity of specimens. After excluding the species where only male or female specimens were used, we conducted ANOVA using PC scores to examine whether there is a statistically significant sexual dimorphism in the size and shape of the skull in these taxa. Our analysis found no significant sex-specific differences in the skull morphology (electronic supplementary material, table S3). For some specimens, especially in the case of extinct Hawaiian honeycreeper species, the sex was unknown. Considering the absence of significant intersexual differences in the skull morphology mentioned above, we analysed skulls of both sexes for the honeycreepers and their relatives while we examined only male specimens for Darwin's finches and their relatives. Information about the specimen ID and sex of each specimen used for GMM analyses is provided in the electronic supplementary material, table S4.

### (b) X-ray microcomputed tomography

High-resolution three-dimensional images of the heads of the specimens were taken using an XRA-002 microCT scanner (X-Tek) available at the Center for Nanoscale Systems at Harvard University, under the condition of 70 kV and 70 µA. Three-dimensional reconstructions were performed with CTPro (Metris) and VGStudio Max 2.0 (Volume Graphics). Landmarks were placed onto each three-dimensional digital image of the skeletons using Amira 5 (FEI). Because skulls of the majority of the species studied can be considered symmetrical (excepting the skulls of the honeycreepers *Loxops caeruleirostris* and *L. coccineus* that have been regarded as more or less asymmetrical) and our research question does not address the evolution of cranial left–right asymmetry, we placed landmarks only on the right side of the skull to streamline our measurements. In cases where the right side of the skeleton was damaged or broken, the landmarks were placed on the left side and the coordinate data were later bilaterally flipped to enable comparison with those for all other specimens in GMM analyses. Originally, a total of 23 landmarks that cover the entire structure were placed on digital images of the skulls (figure 2; electronic supplementary material, table S2) and the coordinates of those landmarks were compared among the representative species of both Darwin's finches and Hawaiian honeycreepers (for detail, see §2c). Unfortunately, the skulls of some Hawaiian honeycreeper species (mainly the extinct/fossil species) were damaged or broken so that placing all 23 landmarks on their skull images was not always possible. Consequently, we reduced the total number of landmarks to 15 for analyses where these specimens were included (these correspond to the original landmarks 1, 2, 3, 4, 5, 6, 15, 16, 17, 18, 19, 20, 21, 22 and 23). Our independent PLS test revealed that the shape covariance matrix of the 15 landmark shape space (LSS) of extant species is strongly equivalent with that found in the original 23 LSS (RV coefficient (0.9904) and permutation test (*p* < 0.0001)), confirming the effectiveness and validity of using a smaller number of key landmarks in our analyses.

### (c) Generalized Procrustes analysis and visualization of morphological variations

Landmark coordinate data were decomposed into size and shape information using generalized Procrustes analysis (GPA) [49]. First, the size of the landmark configuration is represented using natural logarithm centroid size (lnCS), defined as the square root of the sum of squared Euclidian distances from each landmark to the centroid (mean of the configuration of landmark coordinates). Shape, on the other hand, was defined as the residual after size, position and orientation information were excluded [50], where landmark coordinates for each specimen were scaled to unit lnCS, translated to the same centroid, and rotated to minimize the sum of squared distances between equivalent landmarks [51]. The resulting landmark configuration was projected onto Kendall's shape space, as a single point. For the subsequent statistical analyses, the points were projected onto a linear tangent space and shape information was analysed using standard statistics. In the following analyses, the position of each specimen within the shape space was represented as a point in the scatter plots (sub shape spaces) and the shape changes along the respective axes were visualized using three-dimensional deformation of the wireframe connecting the landmarks. These procedures were performed using the MorphoJ 1.70 software [52].

### (d) Evolutionary allometry

When variation in size and shape exists among studied taxa, the size and shape usually covary with each other. The covariation of size and shape along a phylogeny is referred to as evolutionary allometry [53]. For exploring shape variability in landmark configurations after GPA and tangent projection, we decomposed shape variation into the size-related component (evolutionary allometry) and the residual component. We employed multivariate regression of shape variables against lnCS without considering group structures [54]. We chose to use original shape data to increase regression scores, instead of focusing on independent contrasts (IC) [48,55], since IC-based regression considerably decreases the degrees of freedom by summarizing individual shape variation as the species mean. The percentage of regression component was shown in per cent (%). To test whether morphological evolution through adaptive radiation on islands follows the pattern of evolutionary allometry in closely related outgroup taxa to each radiation, we compared the regression lines between the respective songbird groups. Linear regression with lnCS, groups and lnCS × groups interaction as factors was performed between each pair of the groups. The difference between regression slopes was evaluated based on the significance of the lnCS × group interaction. The multiple linear regression (MLR) model for this analysis is as follows:2.1
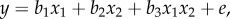
where *y* is the shape vector (Procrustes coordinates), *x*_1_ is lnCS, *x*_2_ is group, *x*_1_*x*_2_ is the interaction between *x*_1_ and *x*_2_, *b* is regression coefficients and *e* is error effect. The MLR model employed lnCS (size: continuous variable) and group (discrete variable) as two explanatory variables and Procrustes coordinates (shape: continuous variable) as an objective variable. The first term denotes the effect of lnCS (size) on shape. Thus, if the coefficient *b*_1_ is significantly deviated from zero, by rejecting the null hypothesis *b*_1_ = 0, there exists allometry (shape change with size change). The second term is the effect of group difference on shape. Thus, in the scatter plot with lnCS on the *x*-axis and regression score (shape) on the *y*-axis as we show in electronic supplementary material, figure S1, the first term is depicted as the slope of regression line and the second term as difference of intercept, respectively. Besides, the third term is the interaction of *x*_1_ and *x*_2,_ which means the effect of group differences on the allometry. If *b*_3_ is significantly deviated from zero and this term is shown effective, the allometry shape changes with size change are varied among groups. This can be understood by modifying formula (2.1) as follows:2.2
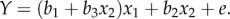
Here, *b*_1_ in parentheses denotes the coefficient of common slope among groups and *b*_3_*x*_2_ is a term for adjustment for group specific slopes. If *b*_3_ is significantly deviated from zero, there is a significant difference of the slopes among groups and the pattern of allometry is not shared. MLR analyses were performed using R 2.14.2 [56].

### (e) Principal component analysis of the whole skull

PCA was performed to reduce a large set of variables to a few dimensions that represent most of the variation [45]. The relative position of the skull of each songbird species was plotted on a scatter plot representing the shape space, for the major axes. Shape changes along the PCs corresponding to their eigenvector were visualized using wireframes. The amount of contribution of each PC axis to overall morphological variation (%) was also calculated. The original configuration with 23 landmarks in three dimensions had 69 degrees of freedom (d.f.). We fit all samples in the same origin, same position and same size (general Procrustes fitting) for skull shape comparison. During this process (translation in three dimensions, rotation in three-dimensional space and scaling), we consume 3 + 3 + 1 d.f. from the original configuration, which finally leaves 62 dimensions (d.f.) for the shape data. In addition to allometry-uncorrected PCA, we performed allometry-corrected PCA as well, by exploring residual shape space to remove the effects of size.

### (f) Neighbour-joining cluster analysis to build a phenogram to show morphological similarity in the skull shape

Similarity of skull shape was measured as the relative distance between the means of two species in multi-dimensional shape space. Mean scores of 77 species in whole-skull PCA were used to calculate interspecies Euclidian distances. Neighbour-joining (NJ) cluster analysis was performed to reconstruct a phenogram, which summarizes relative closeness of skull shape in multi-dimensional space. NJ cluster analysis was performed using Past [57].

### (g) Phylogeny mapping

To describe the evolutionary pattern of shape changes, we mapped phylogeny onto the PCA scatter plots using the squared-changed parsimony method implemented in MorphoJ [52]. Nexus files of the phylogenetic trees were prepared using Mesquite [58]. To test whether phylogeny influences the pattern of morphological variation, a permutation test was conducted (*n* = 10 000).

### (h) Local PCA of the functional subdivisions within the skull

The skulls of songbird species where all 23 landmarks were used were subdivided into six anatomically distinct sectors: upper beak, orbit, palatine, pterygoid, quadrate and adductor chamber (figure 4*a*). PCA was carried out to compare morphology of each subdivision among specimens.

### (i) Partial least-square analysis

The degree of morphological covariation between the whole skull and each sector within the skull (see §2h) was explored using PLS analysis. PLS analysis is based on a singular value decomposition of the matrix of covariances between the two sets of variables [45,50,59]. PLS analyses were conducted using MorphoJ [52].

## 3. Results

### (a) Comparison of the modes and magnitude of skull shape diversification in Darwin's finches and Hawaiian honeycreepers

Morphometric analyses in which the more-inclusive landmark datasets were employed (using the final set of 15 landmarks which allows the extinct Hawaiian honeycreeper species to be analysed) on Darwin's finches, Hawaiian honeycreepers and their respective outgroups, reveal a highly significant degree of allometry (*p* < 0.0001), which explains 12.6% of the variation in shape of the skull (electronic supplementary material, table S5). From the multivariate regression, a larger skull size is found to be associated with a longer and deeper upper beak, relatively smaller orbits, more posteriorly positioned craniofacial hinge, longer, wider and more ventrally oriented palatine bones, and enlargement of the jaw adductor muscle in the antero-posterior direction (electronic supplementary material, figure S1). The slopes of the regression lines for Darwin's finches and Hawaiian honeycreepers are steeper than those for non-Darwin's finch coerebin species, non-Hawaiian honeycreeper fringillid finches, and outgroup taxa (electronic supplementary material, table S5). The slopes and intercepts of Darwin's finches and Hawaiian honeycreepers are statistically significantly different from those of all other included groups (*p* < 0.001). The slope of the regression line of Darwin's finches is steeper than that of the Hawaiian honeycreepers (electronic supplementary material, table S5) and the regression lines for these two lineages are significantly different from one another (*p* < 0.001). Irrespective of the number of the landmarks exploited (whole 23 landmarks or minimum set of 15 landmarks), the results of the analyses of evolutionary allometry are not very different, showing a high level of correlation between the patterns acquired from two independent datasets (electronic supplementary material, figure S2). The patterns of evolutionary allometry for Hawaiian honeycreeper species were slightly different between the trials based on whole 23 landmarks and based on minimum 15 landmarks (electronic supplementary material, figure S2). This is probably because the contribution of the landmarks defining the upper beak to the whole-skull allometry becomes relatively larger in this lineage when only 15 landmarks were considered.

**Figure 3.**
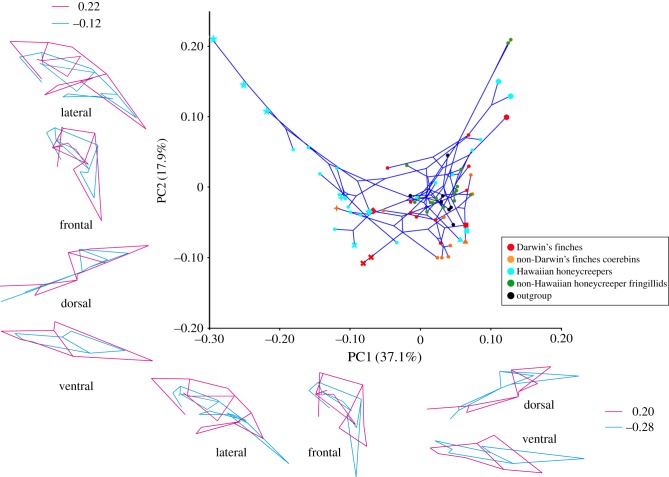
Phylomorphospace of the skulls of Darwin's finches, Hawaiian honeycreepers and other songbird taxa for comparison made using principal components analysis. Sky blue stars, *Drepanis pacifica*, *Vestiaria coccinea* and *Akialoa ellisiana*; sky blue ‘plus’ symbols, *Chlorodrepanis virens*; orange ‘plus’ symbol, *Coereba flaveola*; sky blue ‘cross’ symbol, *Paroreomyza montana*; red ‘cross’ symbol, *Certhidea olivacea* and *C*. *fusca*; sky blue triangle, *Psittirostra psittacea*; orange triangle, *Melopyrrha nigra*; sky blue diamond, *Ciridops anna*; red diamond, *Pinaroloxias inornata*; sky blue square, *Loxioides bailleui*; red square, *Camarhynchus psittacula*; red hexagon, *Geospiza magnirostris*; sky blue hexagons, *Chloridops kona* and *Chloridops wahi*. The lines connecting species positions inside the scatter plot represent phylogenies. Coloured outlines outside of the scatter plot are wireframes for each PC axis to demonstrate the morphological variability of sampled specimens. The scores for blue (negative) and red (positive) wireframes are derived from the negative and positive limits of the scatter plot, respectively.

The PCA of variation in allometry-uncorrected skull shapes reveals that four dimensions (out of 62) explain most (71.8%) of the shape variation among all of the studied species (electronic supplementary material, table S6). In particular, PC1 explains 37.1% of the total variation and mainly describes changes in the length and depth of the beak, depth of the cranium and relative positions of landmarks associated with the adductor chamber (jaw adductor muscle attachment site), and the length and width of bones forming the palate (figure 3). PC2 explains 17.9% of variation and pertains to differences in length and width of the beak, width of the ventral part of the cranium and width of bones forming the palate (figure 3). Shape changes associated with PC3 (11.4%) and PC4 (5.4%) mainly describe changes in the degree of dorsoventral bending of the beak and the proportions of bones forming the palate (electronic supplementary material, figure S3). The result of the PCA after allometry correction is given in the electronic supplementary material, figure S4. Because the pattern of the phylomorphospace is almost identical between the original and allometry-corrected PCAs, hereafter, we shall focus on the results of the original PCA.

Mapping the cranial shapes onto the phylogeny using squared-change parsimony yields a tree length of 0.399 (in units of squared Procrustes distance). The permutation test for a phylogenetic signal in the shape data is highly significant (*p* < 0.0001). The phylomorphospace analysis reveals that the cluster of skull shapes of Darwin's finches and their close relatives (non-Darwin's finch coerebins) is nested within the cluster of Hawaiian honeycreepers with some overlap of PC plots between the two groups that indicates a degree of morphological convergence since both groups evolved such similar features from a morphologically distinct ancestral condition (figure 3; electronic supplementary material, figure S5). Darwin's finches and their relatives tend to occupy a part of the morphospace with moderate PC1 and low to moderate PC2 scores. By contrast, the skull shapes of Hawaiian honeycreepers tend to be distributed along the whole spectrum of the PC1 axis and to have higher PC2 scores. We established that morphological variance in Hawaiian honeycreepers is more than twice as high as that in any other lineages studied (electronic supplementary material, figure S6).

The Hawaiian honeycreeper species featuring elongated and curved bills with preference for nectar feeding or probing for insects (Hawaii mamo (*Drepanis pacifica*), iiwi (*Vestiaria coccinea*), greater akialoa (*Akialoa ellisiana*); sky blue stars in figure 3) occupy a unique domain of morphospace with low PC1 and high PC2 scores. The phylogeny, projected onto the morphospace, also revealed multiple examples of morphological convergence. Such convergence, for example, is observed in the skull shapes of the insectivorous warbler finch (*Certhidea olivacea* and *C*. *fusca*; red ‘cross symbols’ in figure 3) and the Maui alauahio (*Paroreomyza montana*; sky blue ‘cross symbol’ in figure 3), from the Darwin's finch and Hawaiian honeycreeper clades, respectively. Likewise, the skull shape of the large ground finch (*Geospiza magnirostris*; red hexagon in figure 3) in the Galápagos, a species that cracks very hard seeds with its beak, resembles those of two Hawaiian grosbeak species (*Chloridops* spp.; sky blue hexagons in figure 3), one of which was observed in life to also crack very hard seeds [6,15]. Interestingly, morphological convergence in skull shapes is also detected between several species with somewhat dissimilar diet preferences. For example, skull shape of the mostly insectivorous large tree finch (*Camarhynchus psittacula*; red square in figure 3) and that of the palila (*Loxioides bailleui*; sky blue square in figure 3), a mostly plant-eating Hawaiian honeycreeper, are similar to one another [6,60]. Also, convergence of skull shapes was observed between the Cuban bullfinch (*Melopyrrha nigra*; orange triangle in figure 3) and the ou (*Psittirostra psittacea*; sky blue triangle in figure 3), both of which eat fruit and other plant tissues, although the bullfinch appears to emphasize seeds and the ou to emphasize fruit [15,61]. Furthermore, the skull shape of the Cocos finch (*Pinaroloxias inornata*; red diamonds in figure 3) with its insectivorous–frugivorous mixed diet resembles that of the ula-ai-hawane (*Ciridops anna*; sky blue diamonds in figure 3) on Hawaii with an unknown diet. Finally, the skull shape of the strongly nectarivorous bananaquit (*Coereba flaveola*; orange ‘plus symbols’ in figure 3) morphometrically resembles that of the Hawaii amakihi (*Chlorodrepanis virens*; sky blue ‘plus symbols’ in figure 3) with its mixed insectivorous–nectarivorous diet [15,62].

### (b) The contribution of each functional subdivision to whole-skull shape diversification

**Figure 4.**
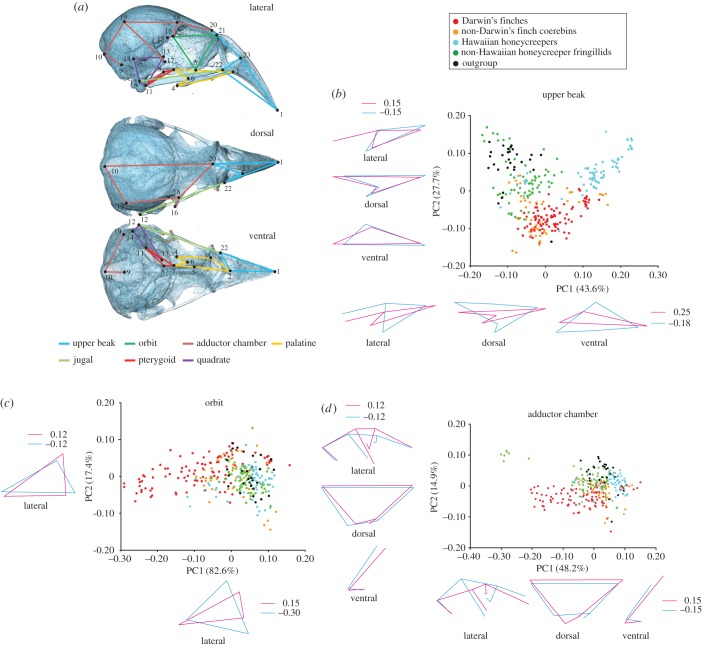
Morphological disparity of each cranial subdivision. (*a*) Partitions of the landmarks corresponding to each cranial functional subdivision: upper beak (sky blue), orbit (dark green), adductor chamber (reddish brown), palatine (orange), jugal (pale green), pterygoid (red) and quadrate (purple). The number of each landmark (figure 2) is provided next to the circles. (*b–d*) Principal component analyses and associated patterns of morphological transformation for the upper beak (*b*), orbit (*c*) and adductor chamber (*d*) among five groups of songbirds: Darwin's finches (red), non-Darwin's finch coerebins (orange), Hawaiian honeycreepers (blue), non-Hawaiian honeycreeper fringillids (green), shared outgroup (black). Each dot represents individual skull specimen.

To understand which part of the skull changed most during the overall morphological diversification in the two songbird lineages that underwent extensive adaptive radiation, we compared the morphology of each subdivision of the skull: upper beak, orbit, palatine, pterygoid, quadrate and adductor chamber (figure 4*a*) among taxa, using PCA. Here, the subset of birds for which the whole 23 landmarks (figure 2) were acquired was analysed. In the case of the upper beak (figure 4*b*), Darwin's finches and their close relatives (i.e. non-Darwin's finch coerebins) occupy a part of the morphospace with moderate PC1 (43.6% of the total variation) and low to moderate PC2 (27.7%) scores, with relatively wider and shorter beaks. By contrast, Hawaiian honeycreepers distribute along the whole spectrum of the PC1 axis and tend to have higher PC2 scores. Therefore, this clade includes species with longer, shallower and narrower beaks as well as species with shorter, deeper and wider beaks. Both non-Hawaiian honeycreeper fringillids (i.e. the taxa indicated with the green branches in figure 1*c*) and shared outgroup taxa (i.e. 7 representative species belonging to the families Estrildidae and Ploceidae) occupy a part of the morphospace with low PC1 and moderate to high PC2 scores, possessing shorter, deeper, wider and more dorsally oriented beaks.

By contrast, in regards to orbit morphology (figure 4*c*), Darwin's finch species occupy the whole spectrum of the PC1 (82.6% of the total variation) axis with moderate PC2 (17.4%) scores. Such scores indicate that this clade contains species with shallower and relatively posteriorly positioned orbits as well as species with deeper and relatively anteriorly positioned orbits. All other songbird groups: non-Darwin's finch coerebins, Hawaiian honeycreepers, non-Hawaiian honeycreeper fringillids and shared outgroup taxa almost overlap to each other in distribution within the morphospace with high PC1 and low to high PC2 scores, indicating possession of relatively more posteriorly located shallower orbits.

In the case of the adductor chamber (figure 4*d*), Darwin's finch species occupy a wide spectrum of the PC1 axis with moderate to high scores, showing a similar distribution to the orbit. This indicates that in this clade there is variation from species with a broader jaw adductor muscle attachment site to species with a smaller attachment site. All other songbird groups overlap in distribution within the morphospace with moderate PC1 (48.2% of the total variation) and low to high PC2 (14.9%) scores, except for two granivorous non-Hawaiian honeycreeper fringillid species: *Coccothraustes* and *Hesperiphona* with low PC1 and high PC2 scores, reflected by their possession of well-developed jaw adductor muscles. The results of PCAs for palatine, pterygoid and quadrate subdivisions, where the morphological disparity among the lineages is indistinct compared with the upper beak, orbit and adductor chamber subdivisions, are provided in the electronic supplementary material, figure S7.

### (c) Covariation between the shape transformation of the whole skull and each of its functional subdivisions

We next evaluated how the shape of the whole skull covaries with that of each cranial subdivision in three groups of birds: (i) Darwin's finches and (ii) Hawaiian honeycreepers, the two groups that underwent extensive adaptive radiation and (iii) all other remaining taxa (i.e. non-Darwin's finch coerebins + non-Hawaiian honeycreeper fringillids + shared outgroup taxa). We used the two-block PLS analysis (table 1). In combined songbird taxa, when excluding Darwin's finches and Hawaiian honeycreepers, there is relatively strong covariation detected between the shape of the whole skull and that of adductor chamber (the RV coefficient is 0.77 (*p* < 0.001)). The shape of the whole skull also covaries with those of the upper beak and the palatine, indicated by moderate RV coefficients (0.62 and 0.65, respectively (*p* < 0.001)). In Darwin's finches, strong covariation is detected between the shape of the whole skull and those of the upper beak, orbit, palatine and adductor chamber (the RV coefficients are 0.76, 0.90, 0.82 and 0.93, respectively (*p* < 0.001)). By contrast, in Hawaiian honeycreepers, strong covariation is only detected between the shape of the whole skull and that of the upper beak (the RV coefficient is 0.94 (*p* < 0.001)). The overall skull shape of this songbird clade only weakly or moderately covaries with the shapes of other cranial subdivisions: the orbit, palatine, pterygoid, quadrate and adductor chamber (the RV coefficients are 0.46, 0.62, 0.17, 0.55 and 0.63, respectively (*p* < 0.001)). Strong covariation between the skull and upper beak is also implied by the fact that the overall skull shape (figure 3) and the beak shape alone (figure 4*b*) produce the same morphospace distribution suggesting the dominance of the beak shape in morphological change of the whole skull. There is evidence for covariation between the upper beak and palatine as the palatine morphospace (electronic supplementary material, figure S7*a*) is similar to the beak morphospace (figure 4*b*).

**Table 1.** The degree of covariation between the shape transformation of the whole skull and each of its functional subdivisions. Scores are RV coefficients.

bird group	upper beak	orbit	palatine	pterygoid	quadrate	adductor chamber
Darwin's finches	0.7619	0.9049	0.8162	0.2856	0.6825	0.9296
Hawaiian honeycreepers	0.9373	0.4550	0.6223	0.1720	0.5480	0.6310
all remaining taxa	0.6219	0.1978	0.6553	0.1223	0.2495	0.7746

### (d) A comparison of the pattern of morphological transformation of the skull within songbird clades that underwent extensive adaptive radiation

**Figure 5.**
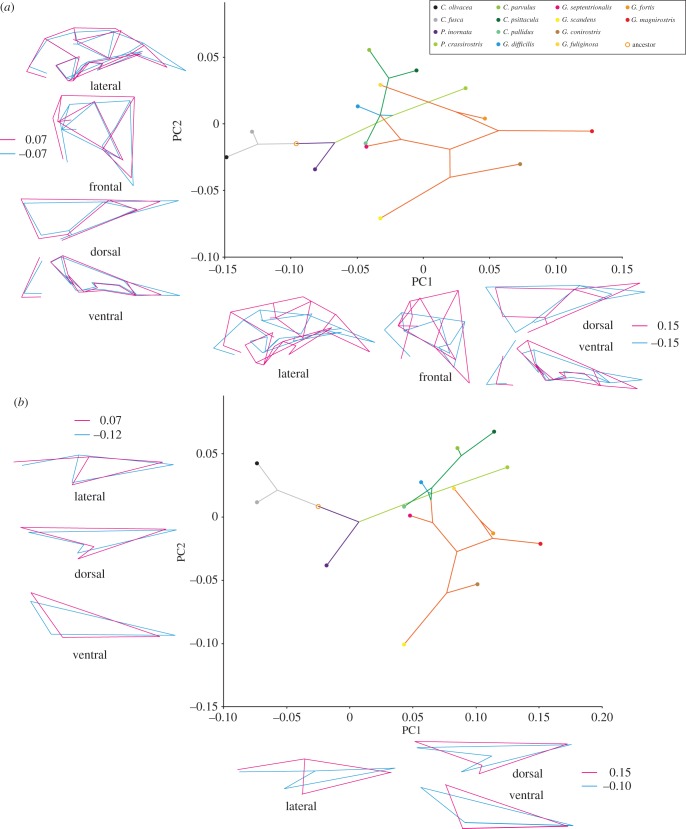
Phylomorphospace of the whole skull and upper beak of Darwin's finch species made using principal components analysis. (*a*) The whole skull (tree length: 0.044; *p*-value: 0.005) and (*b*) the upper beak (tree length: 0.033; *p*-value: <0.0001). PC scores for the mean shape of each species were mapped to the phylogenetic tree from figure 1, using unweighted squared-change parsimony.

**Figure 6.**
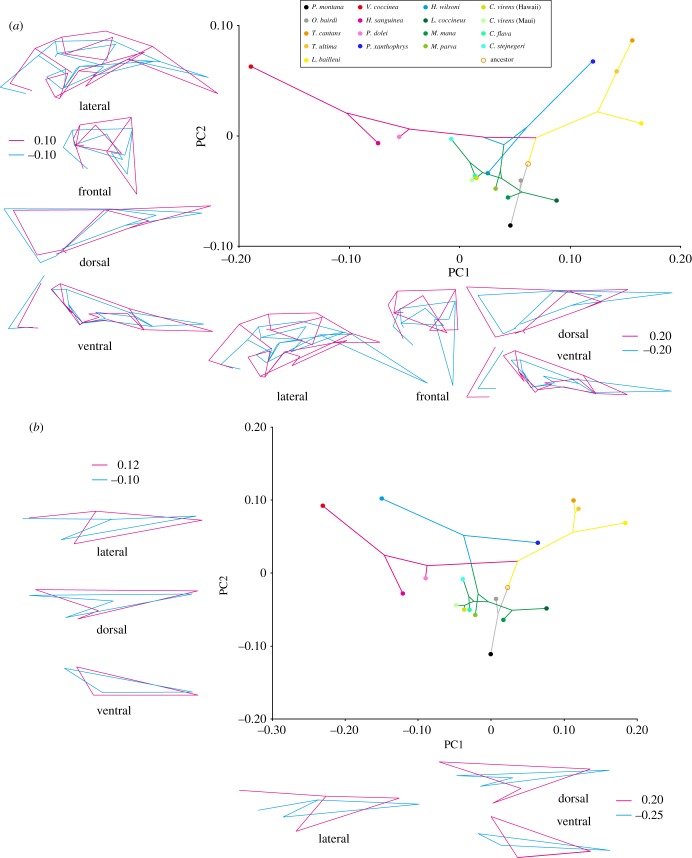
Phylomorphospace of the whole skull and upper beak of Hawaiian honeycreeper species made using principal components analysis. (*a*) The whole skull (tree length: 0.085; *p*-value: 0.0001) and (*b*) the upper beak (tree length: 0.118; *p*-value: 0.0006). PC scores for the mean shape of each species were mapped to the phylogenetic tree from figure 1, using unweighted squared-change parsimony.

To identify both general and unique attributes in the morphological evolution of the skull in songbird clades that underwent adaptive radiation on oceanic islands, we describe and compare patterns of morphological transformation of the skull and upper beak within Darwin's finches and Hawaiian honeycreepers. Considering first the shape of the upper beak, the pattern of its phylomorphospace almost coincides with that for the whole skull in both clades, showing that transformation of the shape of the upper beak makes a large contribution to total transformation of skull shape (RV coefficients are 0.76 in Darwin's finches and 0.94 in Hawaiian honeycreepers; figures 5 and 6).

In Darwin's finches (figure 5), after divergence of the clades containing the warbler finches (*Certhidea*) and Cocos finch (*Pinaroloxias*), skull shape evolves into the form with higher PC1 scores: the skull with deeper and wider upper beak, dorsoventrally higher and medio-laterally wider orbit, shorter, wider and postero-ventrally more bended palatine, and larger as well as more laterally positioned quadrate. However, in the morphospace with relatively higher PC1 scores, the domains for the vegetarian finch (*Platyspiza*), the tree finches (*Camarhynchus*) and the ground finches (*Geospiza*) do not fully overlap. The skull of the genus *Camarhynchus* occupies the part of morphospace with higher PC2 scores, showing that their skulls have relatively shorter and wider upper beak, larger orbit, larger quadrate and broader area for the jaw adductor muscle attachment. By contrast, the skulls of the genus *Geospiza* occupy a morphospace with relatively low PC2 scores, showing that their skulls have relatively longer and narrower upper beak, smaller orbit and a smaller area for the jaw adductor muscle attachment. The skulls of the genus *Platyspiza* occupy the intermediate domain between those for *Camarhynchus* and *Geospiza*, in the morphospace with higher PC1 scores. Interestingly, the skull shapes of some *Geospiza* species (e.g. *G. fuliginosa* and *G. septentrionalis*) show convergence with those of *Camarhynchus*, featuring a skull shape with relatively low PC1 and high PC2 scores. Within the genus *Geospiza*, *G*. *scandens* possesses a unique skull shape occupying an open morphospace with intermediate PC1 and rather low PC2 scores.

Evolutionary allometry of the Darwin's finch skull accounts for a moderate portion of cranial shape variation among the species studied (27.9% of the total variance of shape contrasts is associated with skull size variation). The permutation test for allometry is highly significant (*p* < 0.0001). From the multivariate regression, larger skull size is associated with a longer and deeper upper beak, anterior shift of the position of foramen magnum, postero-dorsal expansion of jaw adductor muscle attachment site, postero-lateral shift of the jugal bone articulation with the quadrate, narrower palatine bones and relatively smaller orbits (electronic supplementary material, table S7 and figure S8). The slope of the regression line for the skulls of ground finches (*Geospiza*) was significantly different from that for the skulls of the tree and vegetarian finch (*Camarhynchus* + *Platyspiza*) group, as well as from that for the skulls of the warbler and Cocos finch (*Certhidea* + *Pinaroloxias*) group (electronic supplementary material, table S7 and figure S8). A combination of the large skull size with a distinct shape has been achieved only by some species belonging to the genus *Geospiza* (electronic supplementary material, figure S8). Our observations are in line with other studies of macro-evolutionary allometry in birds, which reported a generally moderate effect on the overall variation of skull shapes, but do not account for the unusual skull shapes of the outlying taxa [40,44,48].

Within the Hawaiian honeycreepers (figure 6), after divergence of the earliest lineages *Oreomystis* and *Paroreomyza*, that feature a skull shape with moderate PC1 and low PC2 scores, subsequently the skull shape mainly diversifies along two separate directions: the clade that consists of *Telespiza* and *Loxioides* acquires the skull shape with a relatively shorter, wider and deeper upper beak, larger, wider, as well as more anteriorly positioned orbit, more ventrally positioned palatine and pterygoid bones, larger and more laterally positioned quadrate, and broader area for the jaw adductor muscle attachment (high PC1 scores). The other clade consists of *Vestiaria* and *Himatione*, which instead acquires the skull shape with a relatively longer, narrower and shallower upper beak and a more dorsoventrally compressed cranium (low PC1 scores). *Pseudonester* and *Hemignathus* occupy a part of the morphospace between the two aforementioned clades. Interestingly, the skull shape of *Pseudonester* converges with those of *Telespiza*, featuring a similar shorter, deeper, and wider upper beak as well as dorsoventrally more expanded cranium. Although the whole-skull shape of *Hemignathus* is placed near the centre of the morphospace (with both moderate PC1 and PC2), when only upper beak shape is considered it is placed near that of *Vestiaria*, with low PC1 and high PC2 scores, showing dissociation of morphological transformation of the upper beak relative to that of the whole skull. Lastly, a clade that consists of *Chlorodrepanis*, *Loxops*, *Magumma* and *Manucerthia* occupies a rather restricted domain of the morphospace with both moderate PC1 and low to moderate PC2 values. These are placed basally near the root of the phylogeny as well as close to the two earliest diverged Hawaiian honeycreeper taxa (*Oreomystis* and *Paroreomyza*), indicating a resemblance of their skull morphology.

Evolutionary allometry of the Hawaiian honeycreeper skull diversity accounts for a moderate portion of cranial shape variation among species (11.1% of the total variance of shape contrasts is associated with skull size variation). The permutation test indicates that allometry in this group is highly significant (*p* < 0.0001). From the multivariate regression, a larger skull size is associated with a longer and deeper beak, smaller orbits, posterior shift of the position of the craniofacial hinge, longer palatine bones and anterior shift of the position of postero-dorsal attachment site of the jaw adductor muscle (electronic supplementary material, table S8 and figure S9). When we compare the slope of the regression lines for the skulls among five Hawaiian honeycreeper species groups: (i) *Vestiaria* group, (ii) *Pseudonestor* group, (iii) *Loxops* group, (iv) *Melamprosops* group, and (iv) *Telespiza* group, only the angle between the slope for the *Melamprosops* group and that for the *Vestiaria* group, and the angle between the slope for *Melamprosops* group and that for *Loxops* group, are significantly different from one another (electronic supplementary material, table S8 and figure S9). There is no statistically significant difference in the angles between the slopes for all other pairs of Hawaiian honeycreeper species groups.

## 4. Discussion

Applying comparative morphometric analyses, supplemented with molecular phylogenies projected onto a multivariate morphospace, allowed us to uncover major underlying patterns of variation as well as lineage-specific tendencies in skull shape diversification in two clades of birds that have undergone extensive adaptive radiation on oceanic islands. Our study provides a foundation for future investigations into the developmental and molecular mechanisms that have permitted, directed and constrained the observed trends in cranial morphological evolution in these specific bird lineages and help us to better understand the nature of vertebrate adaptive radiations more generally.

### (a) Hawaiian honeycreepers evolved unique and exceptionally diverse cranial shapes

**Figure 7.**
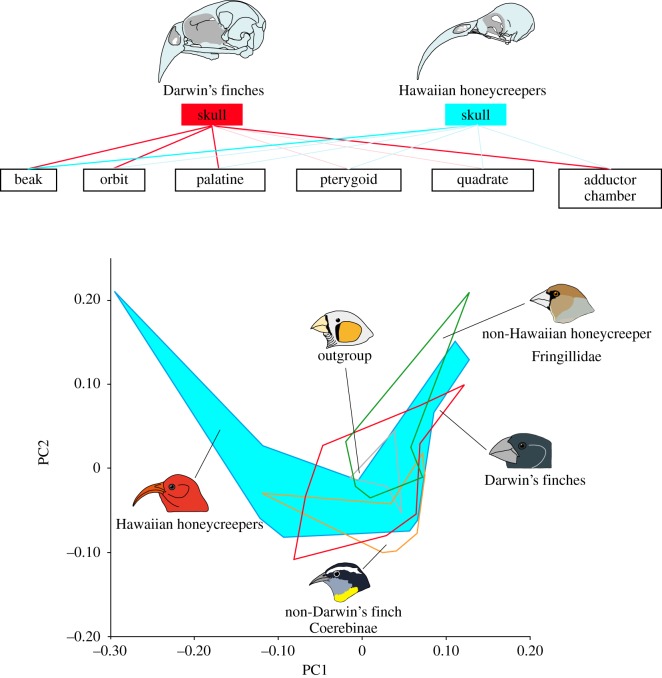
High morphological disparity in the skulls of Hawaiian honeycreepers and its potential intrinsic factors. Darwin's finches showed strong covariation between the shape of the whole skull and those of the upper beak, orbit, palatine and adductor chamber (red thick lines in the top illustration). By contrast, Hawaiian honeycreepers showed strong covariation only between the shape of the whole skull and that of the upper beak (a blue thick line in the top illustration). Such a unique pattern of morphological integration in their skulls probably reflects an altered developmental modularity, which contributed to a high level of disparity of skull morphology observed in Hawaiian honeycreepers (bottom illustration). The polygons in the bottom illustration are based on the result shown in figure 3 and show parts of the morphospace occupied by different groups revealing that Hawaiian honeycreepers (polygon shaded blue) has some of the most diversified and divergent skulls of all species studied.

Adaptive radiation occurs when a lineage becomes decoupled from the normal diversity-dependent controls that regulate macro-evolutionary rates, a release that can occur in response to an extrinsic ecological opportunity and/or to the emergence of novel intrinsic traits that help exploit ecological opportunity (i.e. key innovations) [63], such as the subdigital toe pads in *Anolis* lizards [64] or feathers in birds [65]. In our PCA, the plots of all studied songbird lineages: Darwin's finches, non-Darwin's finch coerebins, Hawaiian honeycreepers, non-Hawaiian honeycreeper fringillids and all further outgroup taxa (i.e. estrildids and ploceids) largely overlapped in the part of the morphospace featuring higher PC1 scores (figures 3 and 7). Interestingly, plots of some Hawaiian honeycreepers uniquely expanded to the morphospace domain with the lower PC1 scores. Strikingly, the overall morphological variance for the skulls in Hawaiian honeycreepers was more than twice as high as in any other lineage (electronic supplementary material, figure S6). This suggests that Hawaiian honeycreepers evolved their unique cranial shapes, many of which are not shared with any other lineage studied here, by uniquely changing the direction of their skull evolution.

The birds with the most distinct cranial shapes are Hawaiian honeycreeper species with a preference for nectarivorous diet, represented by iiwi (*V. coccinea*), Hawaii mamo (*D. pacifica*), and probing for insects in bark or epiphytes, represented by the greater akialoa (*A. ellisiana*; figure 3). They have long and decurved beaks, which are superficially convergent with those in nectarivorous hummingbirds of the New World (Trochilidae, Apodiformes) and nectarivorous sunbirds of the Old World (Nectariniidae, Passeriformes). Previous morphometric studies noted that Hawaiian honeycreepers demonstrate an extraordinary level of diversity in beak morphology compared with both their relatives and unrelated birds inhabiting the same or similar habitats [33,34]. Moreover, several authors previously and independently suggested that this songbird lineage has an intrinsic and unusually high propensity of the beaks to respond to diversifying selection [33,66,67].

In the current study, using advanced three-dimensional geometric morphometrics, we confirmed that the skull shapes of Hawaiian honeycreepers are morphologically more diverse than those of other songbird lineages, with a wider distribution in the PC1 axis of the morphospace (figure 3). Furthermore, our two-block PLS analysis revealed that there is a strong covariation between the skull and upper beak in this songbird lineage expressed as a contribution of the beak to the overall shape (table 1). Covariation between the shapes of the upper beak and the cranium (braincase) has been reported in other lineages of birds such as crows and raptors and it is regarded as basal to the landbird radiation [40,44,48]. Interestingly, in Hawaiian honeycreepers, the other parts of the skull are less strongly coupled with the shape of the whole skull (certainly relative to Darwin's finches; see §4b; table 1). Such results suggest that the high level of disparity in the skull morphology observed in Hawaiian honeycreepers is associated with changes in modularity and integration of individual skull elements, allowing for more evolutionary flexibility to explore the morphospace, as also hypothesized by Bright *et al*. [44].

### (b) Lineage-specific tendencies of skull shape diversification in the adaptive radiation of birds

Recent molecular phylogenetic studies indicated that the ancestors of Darwin's finches and the Hawaiian honeycreepers that colonized each archipelago were, in both cases, small-beaked finch-like birds. Both groups evolved insectivorous forms early in their history [4,8,12,68]. Our GMM analyses of the whole skull revealed that both Darwin's finches on Galápagos and Hawaiian honeycreepers on Hawaii also shared another trend in skull shape evolution in which the ancestral skull configuration (at the root of phylogenies shown in figures 5 and 6) subsequently diverged along two trajectories: (i) towards warbler-/creeper-like forms (with lowest PC1 scores in Darwin's finches and with lowest PC2 score in Hawaiian honeycreepers) and (ii) towards finch/grosbeak-like forms (with higher PC1 scores in Darwin's finches and with higher PC1 and PC2 scores in Hawaiian honeycreepers). Extrinsic factors, such as the type of habitat that was present in each archipelago at the time of colonization, may have favoured insectivorous specialist species in the early phase of diversification in both radiations. It would be interesting to know whether the early evolution of insectivory is a coincidence in these two distinct songbird lineages, or it instead reflects a general rule in adaptive radiations of songbirds.

Our morphometric analysis revealed that skulls with a shallow cranium and shallow, narrow beak, which allow for pecking for small insects, are only achieved by the two closely related warbler finches (*Certhidea*) in the Galápagos. By contrast, the skull shape of the basally diverging ‘creepers’ on Kauai (*Oreomystis bairdi*) and Maui (*P. montana*) islands, which is characterized by a short, narrow, shallow and frontally pointed beak, as well as shallow cranium, was re-acquired by another ‘creeper’ in Hawaii (*Manucerthia mana*) later in the evolution of the Hawaiian honeycreepers. This independent evolution of a skull shape adapted for an insectivorous diet is unique within the Hawaiian honeycreepers and could be explained by the observation that occupation of the niche for small insectivorous birds in the forests of the newest island, Hawaii (in existence for less than 1 Myr) was achieved by the descendents of a late-branching lineage of Hawaiian honeycreepers with a wider distribution in the archipelago (see fig. 2 of [12]) [69]. One likely explanation for the differences in the geographical and phylogenetic distribution of insectivorous species on Galápagos is that the niche for the warbler/creeper-like form was fully occupied by the ‘original’ warbler finch lineage in the Galápagos, perhaps because of relatively short distances between the Galápagos Islands within the archipelago, which helped dispersal of the birds.

In Darwin's finches, skulls with a long and down-curved beak and a shallow and narrow cranium featured by the nectarivorous Hawaiian honeycreeper species (for detail, see §4a) did not evolve from granivorous finch/grosbeak-like forms (figure 5). Our two-block PLS analysis revealed a relatively strong covariation between the shape of the entire skull and those of the upper beak, orbit, palatine and adductor chamber in Darwin's finches (table 1). This implies that the skull of Darwin's finches is a highly integrated structure compared with the Hawaiian honeycreepers (figure 7). It is probable that such levels of integration among the skull modules in Darwin's finches restricted the radiation of morphology and could account for the absence of the skull shapes with a combination of a long and down-curved beak and a shallow and narrow cranium. We conclude that identification of the developmental and molecular mechanisms controlling morphological integration and modularity within the skull will be critical to fully understand the principles of cranial shape evolution in the adaptive radiation of birds.

### (c) Widespread adaptation-related and adaptation-unrelated convergences in skull shapes

Evolutionary convergence is the independent emergence of very similar or identical traits in unrelated evolutionary lineages [70]. The recurrent evolution of convergent forms is a widespread phenomenon in adaptive radiation [71]. Although our PCA revealed a clear phylogenetic signal in variation of cranial skeletal morphology in both Darwin's finches and Hawaiian honeycreepers, multiple cases of morphological convergence were also detected (figure 3; electronic supplementary material, figure S5). For example, skulls with down-curved long beaks and relatively shallower and narrower crania evolved in the Hawaiian species Hawaii mamo (*D. pacifica*) and iiwi (*V. coccinea*), and also independently in the akialoa (*Akialoa* spp.). The mamo and iiwi species consume flower nectar but akialoa feeds on insects in bark and also in epiphytes. Similarly, strong morphological convergence was detected in the skull shapes of warbler finches (*Certhidea*) from the Galápagos and the Maui alauahio (*P. montana*) from Hawaii, both with a specialized insectivorous diet.

A phenotypic resemblance between the Kauai creeper (akikiki, *O. bairdi*) and the Hawaii creeper (*M. mana*) has been reported based on bill shape, the absence of a tubular tongue, tree-creeping foraging and anti-predator mobbing behaviours, and juvenile plumage pattern [15,69,72–75]. Their similarity is so strong that the two species were often treated taxonomically as congeneric or even conspecific in earlier classifications. Although their phenotypic resemblance is now strongly thought to be a product of evolutionary convergence [12,69], our current comparative morphometric study newly reveals that when we focus on the whole skull shape, that of the tree-creeping, insectivorous *O. bairdi* is equally similar to those of the tree-creeping, insectivorous *M. mana* and the insectivorous/nectarivorous anianiau (*Magumma parva*, a species that possesses a tubular tongue and does not exhibit tree-creeping behaviour) based on interspecific Euclidian distances calculated from the shape space (electronic supplementary material, table S9). This evidence that skull shape convergence in these three taxa may have preceded the loss of the tubular tongue and the adoption of tree-creeping foraging behaviour in the lineage leading to *M. mana* lends insight into the evolutionary sequence that produced a remarkable convergence in morphology and behaviour.

Although phenotypic similarity between distantly related taxa has long been considered as strong evidence that natural selection has produced evolutionary adaptations, convergence can occur for reasons unrelated to adaptation and also natural selection [70]. For instance, various types of ‘constraints’ can cause biases in generating or maintaining certain phenotypic outcomes. Because evolution depends on variability, any process that limits the range of available phenotypes will make the repeated evolution of similar features more likely [70]. In our group's recent work, we pointed out the importance of a developmental constraint in the evolution of songbird external beak shape diversity, where the nature of the beak growth programme appears to limit variation to only two major parameters, scaling and shear [32]. The complex patterns of convergences in cranial shapes revealed by the current study suggest that both adaptive natural selection and generative constraints must have played important roles in the morphological divergence of these lineages. Further comparative developmental and multi-disciplinary studies on convergent species will be needed to explain the observed patterns of skull shape and size variation and how similar morphology is generated in unrelated species.

### (d) Significant contribution of allometry to evolution of skull shape variation

Allometry is the dependence of shape on size and tends to be one of the dominant factors of morphological variation [45]. Because size differences among adult animals reflect variation in the extent and rate of growth they have undergone, there is a tight link between adult morphology and ontogeny [45,76]. Allometry can evolve and differ among closely related species [37,38,45,77–80]. Therefore, analysis of evolutionary allometry is indispensable for profound understanding of evolutionary change in growth patterns, which in turn may influence adaptive and functional aspects of adult morphology of animals.

In contrast to a previous study in which the skull shapes of a variety of distantly related lineages of birds, ranging from minute hummingbirds (that weigh less than 5 g) to a gigantic ostrich (that weighs more than 100 kg) were compared [48], we focused on phylogenetically closely related, as well as similarly sized, songbird species (the length of the body has less than a 2.5-fold difference and the body mass has less than an eightfold difference among species; electronic supplementary material, table S1) in this study. Our morphometric analysis revealed a significant contribution of allometry to shape variation in the skull (electronic supplementary material, table S5). Larger skulls tend to have longer and deeper upper beaks, smaller orbits and posteriorly expanded jaw adductor muscle attachment sites. Some trends in allometry that we detected in our songbird samples, such as lengthening and thickening of the upper beak, as well as possession of smaller orbits, in larger species, are shared by other avian lineages [40,44,47]. Moreover, this trend in allometry that larger species have more pronounced upper jaws (beaks) and smaller orbits is even shared by turtles [77], lizards [81], crocodiles [82] and placental mammals [78,79]. Such an allometric trend, which is broadly shared across amniotes, suggests that it might be a ‘rule’ with few known exceptions in the morphological diversification of amniotes.

Interestingly, our analysis of allometry detected certain differences of ontogenetic trajectories among songbird lineages under study. When we compared the slopes of regression lines for skulls, those of Darwin's finches and Hawaiian honeycreepers were steeper than those of non-Darwin's finch coerebins, non-Hawaiian honeycreeper fringillids and shared outgroup taxa (electronic supplementary material, table S5). Our statistical test supported the view that the slopes and intercepts of Darwin's finches and Hawaiian honeycreepers are significantly different from those of all other included groups. Here, a steeper slope indicates that the animals undergo more drastic change of the skull shape through the same amount of growth. We speculate that such alterations of ontogenetic trajectory may relate to rapid diversification of skull morphology in these two songbird lineages that underwent extensive adaptive radiations. Our statistical test also verified that the slope of the regression line of Darwin's finches is steeper than that of Hawaiian honeycreepers and that the regression lines for these two lineages are significantly different from one another (*p* < 0.001; electronic supplementary material, table S5). Both acquiring more data about the ontogeny of Darwin's finches and Hawaiian honeycreepers and identifying the molecular and cellular bases determining ontogenetic trajectories of each group of species will be necessary to better understand the role of development in generation of morphological diversity of songbird skulls.

Lastly, when we compared the slopes of the regression lines for the skull among three groups of Darwin's finches: (i) ground finches including cactus finches (*Geospiza*), (ii) tree finches including woodpecker finch (*Camarhynchus*) + vegetarian finch (*Platyspiza*), and (iii) warbler finches (*Certhidea*) + Cocos finch (*Pinaroloxias*), the slope of *Geospiza* was significantly different from those of other Darwin's finch groups (electronic supplementary material, table S7). Within *Geospiza*, the combination of a large skull size and a distinct shape has been achieved only by ground finches (electronic supplementary material, figure S8). In Darwin's finches, the duration of embryogenesis (between egg-laying and hatching) is not very different among species (all species have a model incubation period of 12 days) [6]. Similarly, the nestling period (between hatching and fledging) does not vary remarkably among species (13–15 days) [6]. It is known that the embryonic growth rate and the relative growth of beaks in embryos and juveniles differ markedly among species; thus, the outstanding challenge is to identify the specific differences in developmental genetic programmes that have led to the remarkable morphological diversity observed in Darwin's finches' skulls [6,83–86]. Such species-specific genetically regulated differences in ontogenetic trajectories, especially in the more specialized forms, must have produced remarkable morphological diversity observed in Darwin's finches' skulls.

## 5. Conclusion

Insights from our three-dimensional morphometric analysis of cranial diversity in two textbook examples of adaptive radiation in avians, Darwin's finches and Hawaiian honeycreepers, offer a new arena for debate about the interplay between the extrinsic (ecological) and intrinsic (genetic) processes, which together generate phenotypic evolutionary diversity. Mapping phylogeny onto the morphospace allowed us to more clearly reveal the accompanying evolutionary phenomena, such as the extent of both morphological divergence and convergence in cranial shapes. We found that the skulls of both Darwin's finches and Hawaiian honeycreepers have diversified greatly beyond and away from the morphological condition of their shared outgroup (representing the more ancestral sparrow-like condition) and their respective close relatives. In particular, Hawaiian honeycreepers show variance in skull shapes that far surpasses any of the other groups studied here, including Darwin's finches. Much of this extreme variation is associated with the highly specialized honeycreeper species feeding on nectar and probing to obtain insects. However, we also detected a significant degree of overlap in the parts of the morphospace occupied by the two clades, showing widespread convergence. Interestingly, many examples of such convergent skull shapes belong to taxa with dissimilar diets. This may indicate the presence of common biomechanical and structural requirements for consuming otherwise distinct food sources (extrinsic constraints) and/or a role for generative (developmental) constraints. Finally, we also measured the influence of allometry on cranial shape changes. We believe that our study will help guide future investigations using methods of biomechanics, comparative genomics, developmental genetics and functional experimentation that are needed to more fully explain the observed evolutionary transitions in skull and beak shape.

## Supplementary Material

Supplementary tables

## Supplementary Material

Supplementary Table 4

## Supplementary Material

Supplementary figures
