# Supplementary material for: Cranial shape evolution in adaptive radiations of birds: comparative morphometrics of Darwin's finches and Hawaiian honeycreepers
Source: Philos Trans R Soc Lond B Biol Sci. 2017 Feb 5;372(1713):20150481. doi: 10.1098/rstb.2015.0481 (PMC5182413; doi:10.1098/rstb.2015.0481)
Supplement: Supplementary tables [file rstb20150481supp1.docx]

**Cranial shape evolution in adaptive radiations of birds:**

**comparative morphometrics of Darwin’s finches and Hawaiian honeycreepers**

**Masayoshi Tokita, Wataru Yano, Helen F. James, Arhat Abzhanov**

***Phil. Trans. R. Soc. B* (2017) doi: 10.1098/rstb.2015.0481**

Supplementary Tables

**Supplementary Table 1.** A list of songbird species analyzed in this study, along with size (length and weight) and diet data for each species. The data are derived from del Hoyo et al. (2014) except for those for extinct Hawaiian honeycreeper species that from Pratt (2005). Symbol for diet: C, vertebrates and their eggs; F, fruits and berries; G, seeds and nuts; I, insects and other invertebrates; N, flower nectar and pollen; S, blood; V, leaves and vegetable matters; ?, no information.

| Species | Length_Ave (cm) | Weight_Ave (g) | Diet |
| --- | --- | --- | --- |
| *Emberiza orix* | 13 | 25·5 | G, I |
| *Vidua macroura* | 11·5 | 15·5 | G, I |
| *Estrilda astrild* | 11·25 | 8·5 | G, I |
| *Lagonosticta senegala* | 9·5 | 9·5 | G |
| *Erythrura prasina* | 15 | 14·7 | G |
| *Taeniopygia guttata* | 10 | 10 | G |
| *Lonchura oryzivora* | 15 | 25·15 | G, I |
| *Geospiza fuliginosa* | 10·25 | 14·5 | G, I, V |
| *Geospiza fortis* | 11·5 | 25 | F, G, I |
| *Geospiza magnirostris* | 15·5 | 33 | F, G, I |
| *Geospiza conirostris* | 14 | 28 | F, I, N, V |
| *Geospiza scandens* | 13 | 22 | F, G, I, N, V |
| *Geospiza septentrionalis* | 11·5 | 16·16 | C, S |
| *Camarhynchus pallidus* | 15 | 25·5 | I |
| *Camarhynchus psittacula* | 13 | 18 | F, I |
| *Camarhynchus parvulus* | 11 | 13·5 | F, I, G, N, V |
| *Geospiza difficilis* | 11·5 | 16·15 | G, I, V |
| *Platyspiza crassirostris* | 16 | 34·5 | F, V |
| *Pinaroloxias inornata* | 12 | 14·55 | F, I, G, N |
| *Certhidea olivacea* | 10 | 10 | I |
| *Certhidea fusca* | 10 | 8 | I |
| *Loxigilla noctis* | 14·75 | 18·25 | F, G, I, N |
| *Tiaris bicolor* | 10·85 | 9·3 | F, G |
| *Tiaris canora* | 11·5 | 7·75 | F, G |
| *Loxipasser anoxanthus* | 10·85 | 11·5 | F, G, I |
| *Loxigilla portoricensis* | 17·75 | 35·75 | F, G, I, V |
| *Melopyrrha nigra* | 14·5 | 13·7 | F, G, I |
| *Loxigilla violacea* | 15·35 | 25·2 | F, G, I, V |
| *Euneornis compestris* | 14 | 16·2 | F, G, I, N |
| *Coereba flaveola* | 10·75 | 20·6 | F, I, N |
| *Tiaris olivacea* | 10·4 | 8 | F, G, I |
| *Melamprosops phaeosoma* | 14 | 25·5 | I, V |
| *Oreomystis bairdi* | 13 | 14·25 | I |
| *Paroreomyza flammea* | 13 | ? | I |
| *Paroreomyza montana* | 11 | 13·9 | I, N |
| *Magumma parva* | 10 | 9·5 | I, N |
| *Loxops caeruleirostris* | 10 | 11 | I |
| *Loxops coccineus* | 10 | 11·1 | I |
| *Manucerthia mana* | 11 | 14·25 | I |
| *Viridonia sagittirostris* | 17 | ? | I, N |
| *Chlorodrepanis virens* | 11 | 13·35 | F ,I, N |
| *Chlorodrepanis stejnegeri* | 11 | 17·25 | F ,I, N |
| *Chlorodrepanis flava* | 11 | 13·9 | F ,I, N |
| *Pseudonestor xanthophrys* | 14 | 25·45 | F ,I, N |
| *Psittirostra psittacea* | 17 | ? | F, V |
| *Akialoa ellisiana* | 19 | ? | I, N |
| *Akialoa obscura* | 17 | ? | I, N |
| *Hemignathus wilsoni* | 14 | 28·4 | I, N |
| *Ciridops anna* | 11 | ? | ? |
| *Palmeria dolei* | 18 | 28·75 | I, N |
| *Himatione sanguinea* | 13 | 16 | I, N |
| *Drepanis pacifica* | 23 | ? | I, N |
| *Vestiaria coccinea Xestospiza conica Rhodacanthis flaviceps* | 15  ? 19 | 19·9  ?  ? | I, N  I (?)  V |
| *Chloridops kona Chloridops wahi Loxioides bailleui Telespiza persecutrix Telespiza ultima* | 15  ? 19  ?  17 | ?  ? 38·1  ? 24·5 | V  V I, V G (?)  C, F, G, I, V |
| *Telespiza cantans* | 19 | 34·4 | C, F, G, I, V |
| *Carpodacus erythrinus* | 14 | 26 | F, G, I, V |
| *Leucosticte arctoa* | 16 | 35 | F, G, I, V |
| *Leucosticte brandti Pyrrhula pyrrhula Pinicola enucleator Haemorhous cassinii Haemorhous purpureus Haemorhous mexicanus Chloris sinica*  *Carduelis carduelis* | 17·75  15·25  22  15·5  14  13·75  13·25  12 | 30  29·5  59·5  29·1  22·95  22·25  19·5  16·75 | G, V  F, G, I, V  F, G, I, V  F, G, I, V  F, G, I, N, V  F, G, I, V  G, I F, G, I, V |
| *Serinus canaria* | 13 | 17·5 | F, G, I, V |
| *Spinus pinus* | 12 | 14·5 | F, G, I, V |
| *Spinus psaltria* | 10 | 9·75 | F, G, I, V |
| *Coccothraustes vespertinus* | 19·75 | 61·4 | F, G, I, V |
| *Coccothraustes coccothraustes* | 17 | 59 | F, G, I, V |
| *Fringilla coelebs* | 16 | 23 | G, I, V |

**Supplementary Table 2.** Definitions of the landmarks used on the skull.

Number Definition

1. The tip of the upper beak
2. The ventro-medial point of the caudal edge of the premaxilla bone
3. The point where the rostro-lateral edge of the palatine bone meets the premaxilla bone
4. The caudal tip of the *processus transpalatinus* of the palatine bone
5. Maximum of curvature at the rostral edge of the *fossa ventralis* of the palatine bone
6. Maximum of curvature at the caudal edge of the *fossa ventralis* of the palatine bone
7. Maximum of curvature at the rostro-lateral surface of the medial articulation (with the parasphenoid bone) of the pterygoid bone
8. The most caudo-ventral point of the medial articulation (with the parasphenoid bone) of the pterygoid bone
9. The subsidence just anterior to the *condylus occipitalis*
10. The most dorsal point of the foramen magnum
11. Ventral surface of the articulation boundary between the pterygoid bone and the quadrate bone
12. The lateral tip of the posterior articulation (with the quadrate) of the jugal bone
13. The tip of the *processus orbitalis quadrati* of the quadrate bone
14. The tip of the *processus suprameaticus* of the quadrate bone
15. The ventro-medial margin of the *processus postorbitalis*
16. The tip of the *processus postorbitalis*
17. The most rostral tip of the *processus zygomaticus*
18. The most rostro-dorsal point of the *crista temporalis*
19. A point on the crista temporalis that is parallel to the laterally-projected ridge (crista) of the *processus postorbitalis* ("Cr4" in Genbrugge *et al.* 2012 *J*. *Anat*.)
20. The mid-point of the cranio-facial hinge
21. The most lateral point of the *processus antorbitalis*
22. The point where the most rostro-lateral edge of the jugal bar meets the upper beak (the *processus maxillaris praemaxillae*)
23. The maximum curvature at the rostral end of the external nares

**Supplementary Table 3.** Procrustes ANOVA for both centroid size (lnCS) and shape (SH) of the skulls of Hawaiian honeycreepers. Sums of squares (SS) and mean squares (MS) are in units of Procrustes distances (dimensionless).

|  | | SS | MS | df | F | *p* (param.) |
| --- | --- | --- | --- | --- | --- | --- |
| lnCS | Sex | 6.068751 | 6.068751 | 1 | 0.18 | 0.676 |
|  | Individual | 3662.785141 | 34.554577 | 106 |  |  |
| SH | Sex | 0.00973651 | 0.000157041 | 62 | 0.52 | 0.9993 |
|  | Individual | 1.98648752 | 0.000302265 | 6572 |  |  |

**Supplementary Table 4.** A list of skeletal specimens analyzed in this study, along with information about catalogue number and sex (male or female). Provided as a separate file.

**Supplementary Table 5.** Statistics about evolutionary allometry in the skulls of all taxa analyzed.

| Sums of squares (SS) |  |
| --- | --- |
| Total SS: Predicted SS: Residual SS: | 6.52705044  0.82430896  5.70274148 |

% predicted: 12.63%

| Permutation test against the null hypothesis of independence | |
| --- | --- |
| Number of randomization rounds P-value | 10000  <0.0001 |

|  | DF | non-DF coerebins | HHC | non-HHC fringillids | Outgroup |
| --- | --- | --- | --- | --- | --- |
| Intercept | -1.42034 | -0.70114 | -1.02633 | -0.67907 | -0.43285 |
| Slope | 0.426885 | 0.203348 | 0.311195 | 0.20838 | 0.127748 |
| Total SS | 1.1856561 | 0.48973143 | 1.9285123 | 0.92240516 | 0.21938416 |
| Predicted SS | 0.53660522 | 0.09443586 | 0.29640118 | 0.19453816 | 0.02659317 |
| Residual SS | 0.64905088 | 0.39529557 | 1.63211112 | 0.727867 | 0.19279099 |
| % predicted | 45.26% | 19.28% | 15.37% | 21.09% | 12.12% |
| P-value | <0.0001 | <0.0001 | <0.0001 | <0.0001 | 0.0005 |

|  | | Difference between the slopes | | | | |
| --- | --- | --- | --- | --- | --- | --- |
|  |  | DF | non-DF coerebins | HHC | non-HHC fringillids | Outgroup |
|  | Coefficient  of Slope | 0.426885 | 0.203348 | 0.311195 | 0.20838 | 0.127748 |
| DF | 0.426885 | 0 | 0.223537 | 0.115691 | 0.218506 | 0.299137 |
| non-DF coerebins | 0.203348 | -0.22354 | 0 | -0.10785 | -0.00503 | 0.0756 |
| HHC | 0.311195 | -0.11569 | 0.107846 | 0 | 0.102815 | 0.183446 |
| non-HHC fringillids | 0.20838 | -0.21851 | 0.005031 | -0.10281 | 0 | 0.080631 |
| Outgroup | 0.127748 | -0.29914 | -0.0756 | -0.18345 | -0.08063 | 0 |

|  | | Pr(>\|t\|) | | | | |
| --- | --- | --- | --- | --- | --- | --- |
|  |  | DF | non-DF coerebins | HHC | non-HHC fringillids | Outgroup |
|  | Coefficient  of Slope | 0.426885 | 0.203348 | 0.311195 | 0.20838 | 0.127748 |
| DF | 0.426885 |  | 0 | 0 | 0 | 0 |
| non-DF coerebins | 0.203348 | 0 |  | 0.007 | 0.896 | 0.208 |
| HHC | 0.311195 | 0 | 0.007 |  | 0.006 | 0.004 |
| non-HHC fringillids | 0.20838 | 0 | 0.896 | 0.006 |  | 0.255 |
| Outgroup | 0.127748 | 0 | 0.208 | 0.004 | 0.255 |  |

**Supplementary Table 6.** PCA of variation among the shapes of species means for the original (allometry-uncorrected) shape.

|  | Eigenvalues | % Total variance | accumulation of % Total variance |  |
| --- | --- | --- | --- | --- |
| 1 | 0.00575891 | 37.104 | 37.104 |  |
| 2 | 0.00277681 | 17.89 | 54.994 |  |
| 3 | 0.00176803 | 11.391 | 66.385 |  |
| 4 | 0.00083451 | 5.377 | 71.762 |  |
| 5 | 0.00053618 | 3.455 | 75.216 |  |
| 6 | 0.00046213 | 2.977 | 78.194 |  |
| 7 | 0.00043167 | 2.781 | 80.975 |  |
| 8 | 0.0003468 | 2.234 | 83.209 |  |
| 9 | 0.00025097 | 1.617 | 84.826 |  |
| 10 | 0.00020594 | 1.327 | 86.153 |  |
| 11 | 0.00019281 | 1.242 | 87.395 |  |
| 12 | 0.00015721 | 1.013 | 88.408 |  |
| 13 | 0.00014999 | 0.966 | 89.374 |  |
| 14 | 0.00014614 | 0.942 | 90.316 |  |
| 15 | 0.00013582 | 0.875 | 91.191 |  |
| 16 | 0.00010354 | 0.667 | 91.858 |  |
| 17 | 0.00009604 | 0.619 | 92.477 |  |
| 18 | 0.0000799 | 0.515 | 92.992 |  |
| 19 | 0.00007809 | 0.503 | 93.495 |  |
| 20 | 0.00007409 | 0.477 | 93.972 |  |
| 21 | 0.00007088 | 0.457 | 94.429 |  |
| 22 | 0.00006224 | 0.401 | 94.83 |  |
| 23 | 0.00005775 | 0.372 | 95.202 |  |
| 24 | 0.00005539 | 0.357 | 95.559 |  |
| 25 | 0.00004838 | 0.312 | 95.87 |  |
| 26 | 0.0000468 | 0.302 | 96.172 |  |
| 27 | 0.00004609 | 0.297 | 96.469 |  |
| 28 | 0.00003921 | 0.253 | 96.721 |  |
| 29 | 0.00003601 | 0.232 | 96.953 |  |
| 30 | 0.00003468 | 0.223 | 97.177 |  |
| 31 | 0.00003203 | 0.206 | 97.383 |  |
| 32 | 0.00002935 | 0.189 | 97.572 |  |
| 33 | 0.00002821 | 0.182 | 97.754 |  |
| 34 | 0.00002624 | 0.169 | 97.923 |  |
| 35 | 0.00002498 | 0.161 | 98.084 |  |
| 36 | 0.00002307 | 0.149 | 98.233 |  |
| 37 | 0.0000212 | 0.137 | 98.369 |  |
| 38 | 0.00002081 | 0.134 | 98.503 |  |
| 39 | 0.00001861 | 0.12 | 98.623 |  |
| 40 | 0.00001782 | 0.115 | 98.738 |  |
| 41 | 0.00001769 | 0.114 | 98.852 |  |
| 42 | 0.0000156 | 0.101 | 98.953 |  |
| 43 | 0.00001548 | 0.1 | 99.052 |  |
| 44 | 0.00001429 | 0.092 | 99.144 |  |
| 45 | 0.00001323 | 0.085 | 99.23 |  |
| 46 | 0.00001232 | 0.079 | 99.309 |  |
| 47 | 0.00001104 | 0.071 | 99.38 |  |
| 48 | 0.00001087 | 0.07 | 99.45 |  |
| 49 | 0.00001007 | 0.065 | 99.515 |  |
| 50 | 0.00000942 | 0.061 | 99.576 |  |
| 51 | 0.00000936 | 0.06 | 99.636 |  |
| 52 | 0.00000833 | 0.054 | 99.69 |  |
| 53 | 0.00000798 | 0.051 | 99.741 |  |
| 54 | 0.00000748 | 0.048 | 99.789 |  |
| 55 | 0.00000674 | 0.043 | 99.833 |  |
| 56 | 0.00000595 | 0.038 | 99.871 |  |
| 57 | 0.00000511 | 0.033 | 99.904 |  |
| 58 | 0.00000427 | 0.028 | 99.931 |  |
| 59 | 0.00000363 | 0.023 | 99.955 |  |
| 60 | 0.00000335 | 0.022 | 99.976 |  |
| 61 | 0.00000193 | 0.012 | 99.989 |  |
| 62 | 0.00000173 | 0.011 | 100 |  |

**Supplementary Table 7.** Statistics about evolutionary allometry in the skulls of Darwin's finches.

| Sums of squares (SS) |  |
| --- | --- |
| Total SS: Predicted SS: Residual SS: | 1.96310345  0.54800119  1.41510226 |

% predicted: 27.92%

| Permutation test against the null hypothesis of independence | |
| --- | --- |
| Number of randomization rounds P-value | 10000  <0.0001 |

|  | *Geospiza* | *Certhidea*/*Pinarol*  *oxias* | *Camarhynchus*/*Platyspi*  *za* | non-DF coerebins | Outgroup |
| --- | --- | --- | --- | --- | --- |
| Intercept | -1.40881 | -0.85963 | -0.8053 | -0.99806 | -0.53808 |
| Slope | 0.394495 | 0.243793 | 0.221722 | 0.279478 | 0.145616 |

|  | | Difference between the slopes | | | | |
| --- | --- | --- | --- | --- | --- | --- |
|  |  | *Geospiza* | *Certhidea*/*Pinarol*  *oxias* | *Camarhynchus*/*Platyspi*  *za* | non-DF coerebins | Outgroup |
|  | Coefficient  of Slope | 0.394 | 0.244 | 0.222 | 0.279 | 0.146 |
| *Geospiza* | 0.394 | 0 | 0.151 | 0.173 | 0.115 | 0.249 |
| *Certhidea*/*Pinaroloxias* | 0.244 | 0.1507 | 0 | 0.022 | -0.036 | 0.098 |
| *Camarhynchus*/*Platyspiza* | 0.222 | 0.17277 | 0.022 | 0 | 0.058 | 0.076 |
| non-DF coerebins | 0.279 | 0.11502 | -0.036 | 0.05776 | 0 | 0.134 |
| Outgroup | 0.146 | 0.24888 | 0.098 | 0.07611 | 0.13386 | 0 |

|  | | Pr(>\|t\|) | | | | |
| --- | --- | --- | --- | --- | --- | --- |
|  |  | *Geospiza* | *Certhidea*/*Pinarol*  *oxias* | *Camarhynchus*/*Platyspi*  *za* | non-DF coerebins | Outgroup |
|  | Coefficient  of Slope | 0.394 | 0.244 | 0.222 | 0.279 | 0.146 |
| *Geospiza* | 0.394 |  | 0.014 | 0 | 0 | 0 |
| *Certhidea*/*Pinaroloxias* | 0.244 | 0.014 |  | 0.656 | 0.673 | 0.02 |
| *Camarhynchus*/*Platyspiza* | 0.222 | 0 | 0.656 |  | 0.105 | 0.02 |
| non-DF coerebins | 0.279 | 0 | 0.673 | 0.105 |  | 0.009 |
| Outgroup | 0.146 | 0 | 0.02 | 0.02 | 0.009 |  |

**Supplementary Table 8.** Statistics about evolutionary allometry in the skulls of Hawaiian honeycreepers.

| Sums of squares (SS) |  |
| --- | --- |
| Total SS: Predicted SS: Residual SS: | 4.05124314  0.45018349  3.60105966 |

% predicted: 11.11%

| Permutation test against the null hypothesis of independence | |
| --- | --- |
| Number of randomization rounds P-value | 10000  <0.0001 |

|  | *Vestiaria* group (Red) | *Pseudonestor*  group (Dark green) | *Loxops* group (Pink) | *Melamprosops*  group (Blue) | *Telespiza* group  (Yellow) | non-HCC  fringillids (Right green) | Outgroup  (Black) |
| --- | --- | --- | --- | --- | --- | --- | --- |
| Intercept | -1.009 | -1.772 | -0.908 | -0.14 | -1.119 | -0.605 | -0.548 |
| Slope | 0.327 | 0.511 | 0.283 | 0.033 | 0.333 | 0.179 | 0.157 |

|  | | Difference between the slopes | | | | | | |
| --- | --- | --- | --- | --- | --- | --- | --- | --- |
|  |  | *Vestiaria* group (Red) | *Pseudonestor*  group (Dark green) | *Loxops* group (Pink) | *Melamprosops*  group (Blue) | *Telespiza* group  (Yellow) | non-HCC  fringillids (Right green) | Outgroup  (Black) |
|  | Coefficient  of Slope | 0.327 | 0.511 | 0.283 | 0.033 | 0.333 | 0.179 | 0.157 |
| *Vestiaria* (Red) | 0.327 | 0 | -0.184 | 0.044 | 0.294 | -0.006 | 0.148 | 0.17 |
| *Pseudonestor* (Dark green) | 0.511 | 0.184 | 0 | 0.228 | 0.478 | 0.178 | 0.332 | 0.354 |
| *Loxops* (Pink) | 0.283 | -0.044 | -0.228 | 0 | 0.25 | -0.05 | 0.104 | 0.126 |
| *Melamprosops* (Blue) | 0.033 | -0.294 | -0.478 | -0.25 | 0 | -0.3 | -0.146 | -0.124 |
| *Telespiza* (Yellow) | 0.333 | 0.006 | -0.178 | 0.05 | 0.3 | 0 | 0.154 | 0.176 |
| non-HCC fringillids (Right  green) | 0.179 | -0.148 | -0.332 | -0.104 | 0.146 | -0.154 | 0 | 0.022 |
| Outgroup (Black) | 0.157 | -0.17 | -0.354 | -0.126 | 0.124 | -0.176 | -0.022 | 0 |

|  | | Pr(>\|t\|) | | | | | | |
| --- | --- | --- | --- | --- | --- | --- | --- | --- |
|  |  | *Vestiaria* group (Red) | *Pseudonestor*  group (Dark green) | *Loxops* group (Pink) | *Melamprosops*  group (Blue) | *Telespiza* group  (Yellow) | non-HCC  fringillids (Right green) | Outgroup  (Black) |
|  | Coefficient  of Slope | 0.327 | 0.511 | 0.283 | 0.033 | 0.333 | 0.179 | 0.157 |
| *Vestiaria* (Red) | 0.327 |  | 0.314 | 0.395 | 0.043 | 0.942 | 0.016 | 0.001 |
| *Pseudonestor* (Dark green) | 0.511 | 0.314 |  | 0.087 | 0.224 | 0.403 | 0.132 | 0.019 |
| *Loxops* (Pink) | 0.283 | 0.395 | 0.087 |  | 0.004 | 0.487 | 0.191 | 0.004 |
| *Melamprosops* (Blue) | 0.033 | 0.043 | 0.224 | 0.004 |  | 0.065 | 0.501 | 0.218 |
| *Telespiza* (Yellow) | 0.333 | 0.942 | 0.403 | 0.487 | 0.065 |  | 0.199 | 0.021 |
| non-HCC fringillids (Right  green) | 0.179 | 0.016 | 0.132 | 0.191 | 0.501 | 0.199 |  | 0.712 |
| Outgroup (Black) | 0.157 | 0.001 | 0.019 | 0.004 | 0.218 | 0.021 | 0.712 |  |

**Supplementary Table 9.** Euculidian distances among the three species of Hawaiian honeycreepers and an outgroup taxon (the medium ground finch, *Geospiza fortis*)

| The distance based on only PC1 and PC2 axes | Manucerthia mana | Magumma parva | Oreomystis bairdi | Geospiza fortis |
| --- | --- | --- | --- | --- |
| Manucerthia mana | 0 | 0.004715 | 0.014951 | 0.180252 |
| Magumma parva |  | 0 | 0.011571 | 0.175538 |
| Oreomystis bairdi |  |  | 0 | 0.168449 |
| Geospiza fortis |  |  |  | 0 |

| The distance based on PC1, PC2, PC3 and PC4  axes | Manucerthia mana | Magumma parva | Oreomystis bairdi | Geospiza fortis |
| --- | --- | --- | --- | --- |
| Manucerthia mana | 0 | 0.026615 | 0.024099 | 0.196662 |
| Magumma parva |  | 0 | 0.041444 | 0.203337 |
| Oreomystis bairdi |  |  | 0 | 0.179815 |
| Geospiza fortis |  |  |  | 0 |

| The distance based on all 62 PC axes | Manucerthia mana | Magumma parva | Oreomystis bairdi | Geospiza fortis |
| --- | --- | --- | --- | --- |
| Manucerthia mana | 0 | 0.074141 | 0.051802 | 0.21066 |
| Magumma parva |  | 0 | 0.076599 | 0.208237 |
| Oreomystis bairdi |  |  | 0 | 0.194063 |
| Geospiza fortis |  |  |  | 0 |
