# Supplementary material for: Cranial shape evolution in adaptive radiations of birds: comparative morphometrics of Darwin's finches and Hawaiian honeycreepers
Source: Philos Trans R Soc Lond B Biol Sci. 2017 Feb 5;372(1713):20150481. doi: 10.1098/rstb.2015.0481 (PMC5182413; doi:10.1098/rstb.2015.0481)

Cranial shape evolution in adaptive radiations of birds: comparative morphometrics of Darwin's finches and Hawaiian honeycreepers

Masayoshi Tokita, Wataru Yano, Helen F. James, Arhat Abzhanov

***Phil. Trans. R. Soc. B* (2017) doi: 10.1098/rstb.2015.0481**

Supplementary Figures

Supplementary Figure 1. Evolutionary allometry of the skull of all species under study (Darwin's finches and Hawaiian honeycreepers, as well as their relatives and outgroup taxa). Evolutionary allometry of skull shape, based on multivariate regression of shape on log-transformed centroid size. The wireframe illustrations show the shapes expected for changes by −0.10 and +0.20 units of log-transformed centroid size from the mean shape.

Supplementary Figure 2. Comparison of the results of allometry analyses of the specimens with all 23 landmarks and the specimens with minimum 15 landmarks (a) Evolutionary allometry among the specimens with all 23 landmarks on their skulls. (b) Evolutionary allometry among the specimens with minimum 15 landmarks on their skulls. (c) Correlation between the regressions for the dataset based on all 23 landmarks and that based on minimum 15 landmarks. RV coefficient for all taxa and for each lineage of songbirds were shown below the scatter plot.

Supplementary Figure 3. Phylomorphospace of the skull of all species under study (Darwin's finches and Hawaiian honeycreepers, as well as their relatives and outgroup taxa). Phylomorphospace (PC3 vs. PC4) of the skull shape made using principal components analysis. PC scores for the mean shape of each species were mapped to the phylogenetic tree given in Fig. 1, using unweighted squared change parsimony. The wireframe illustrations show the extreme cranial shapes representing the positive and negative end of each axis.

Supplementary Figure 4. Allometry-corrected phylomorphospace of the skull of all species under study (Darwin's finches and Hawaiian honeycreepers, as well as their

relatives and outgroup taxa). (a) Phylomorphospace (PC1 vs. PC2) of skull shape corrected for evolutionary allometry, made using principal components analysis (tree length: 0.352; P-value: <0.0001). Sky blue stars, *Drepanis pacifica*, *Vestiaria coccinea*, and *Akialoa ellisiana*; sky blue "+"s, *Chlorodrepanis virens*; orange "+", *Coereba flaveola*; sky blue "X", *Paroreomyza montana*; red "X", *Certhidea olivacea*; sky blue triangle, *Psittirostra psittacea*; sky blue square, *Loxioides bailleui*; red square, *Camarhynchus psittacula*; orange square, *Melopyrrha nigra*. **(b)** Phylomorphospace (PC3 vs. PC4). PC scores for the mean shape of each species were mapped to the phylogenetic tree given in Fig. 1, using unweighted squared change parsimony. The wireframe illustrations show the extreme cranial shapes representing the positive and negative end of each axis.

Supplementary Figure 5. A phenogram using neighbor-joining cluster analysis of whole skull morphology of 77 songbird species.

Supplementary Figure 6. Comparison of morphological variance between the songbird lineages under study. In skull shapes, the variances within Hawaiian honeycreepers are more than twice as high as those within other lineages. The values of morphological variance are almost equivalent between Darwin's finches and non-Darwin's finch Coerebinae. Abbreviation: DF, Darwin's finches; HHC, Hawaiian honeycreepers.

Supplementary Figure 7. Morphological disparity of the palatine, pterygoid, and quadrate bones. (a-b) Principal component analyses and associated patterns of morphological transformation of the palatine (a, PC1 vs PC2; b, PC3 vs PC4) among five groups of songbirds: Darwin's finches (red), non-Darwin's finch coerebins (orange), Hawaiian honeycreepers (blue), non-Hawaiian honeycreeper fringillids (green), shared outgroup (black). Each dot represents individual skull specimen. (c) Principal component analysis and associated patterns of morphological transformation of the pterygoid (PC1 vs PC2). (d-e) Principal component analyses and associated patterns of morphological transformation of the quadrate (d, PC1 vs PC2; e, PC3 vs PC4).

Supplementary Figure 8. Evolutionary allometry of the skull in Darwin's finches. Evolutionary allometry of skull shape, based on multivariate regression of shape on log-transformed centroid size. The regression score is the shape variable that has the direction of the regression vector in shape space, and its relationship to log-transformed centroid size

indicates the strength of allometry. The wireframe illustrations show the shapes expected for changes by −0.10 and +0.15 units of log-transformed centroid size from the mean shape.

Supplementary Figure 9. Evolutionary allometry of the skull in Hawaiian honeycreepers. Evolutionary allometry of skull shape, based on multivariate regression of shape on log-transformed centroid size. The regression score is the shape variable that has the direction of the regression vector in shape space, and its relationship to log-transformed centroid size indicates the strength of allometry. The wireframe illustrations show the shapes expected for changes by −0.10 and +0.25 units of log-transformed centroid size from the mean shape.

Supplementary Figure 1

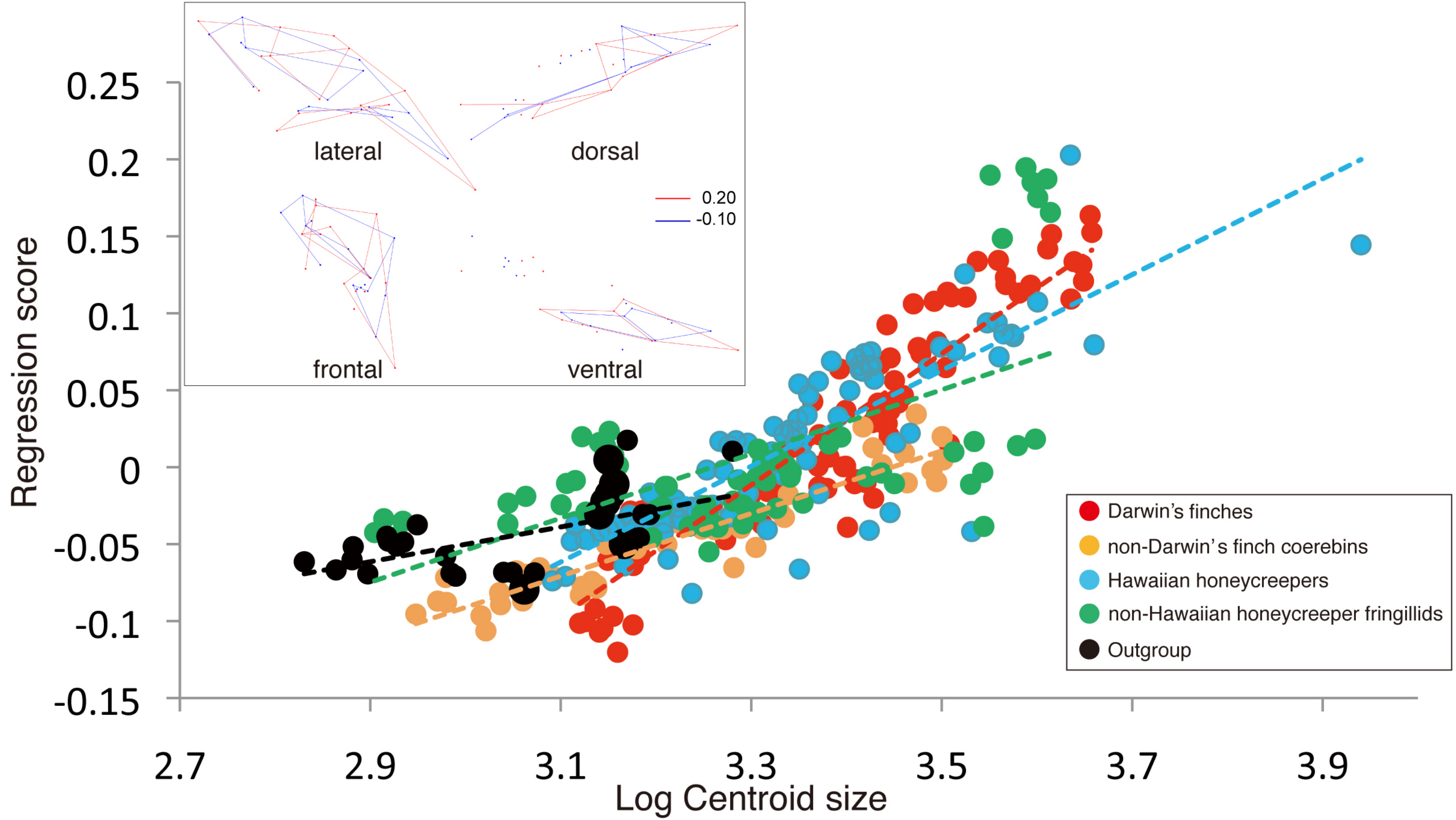

Supplementary Figure 2

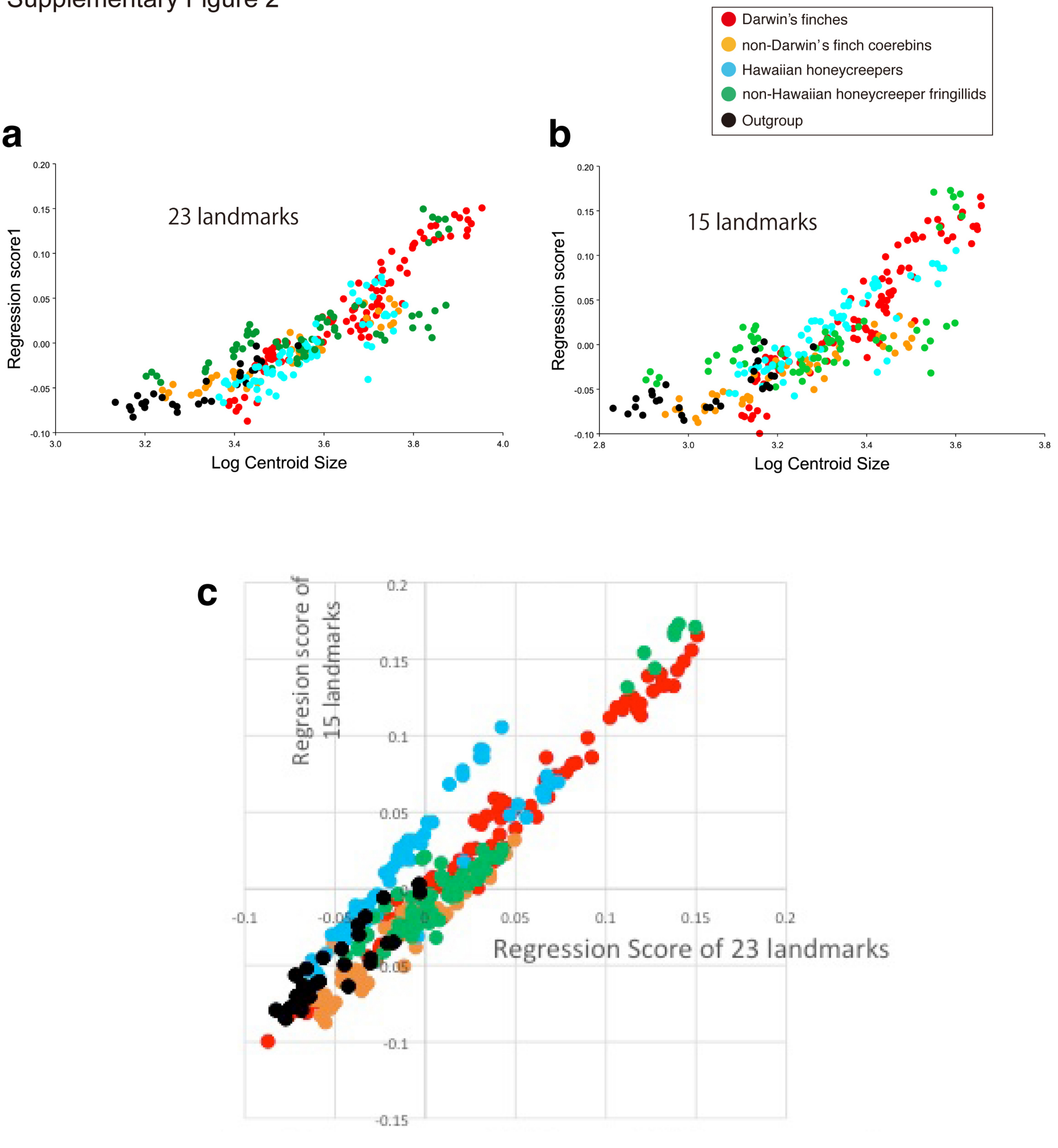

	All taxa	DF	non-DF coerebins	HHC	non-HHC fringillids	Outgroup
RV coefficient	0.946	0.99	0.905	0.897	0.967	0.898
R^2	0.895	0.979	0.82	0.804	0.936	0.806

Supplementary Figure 3

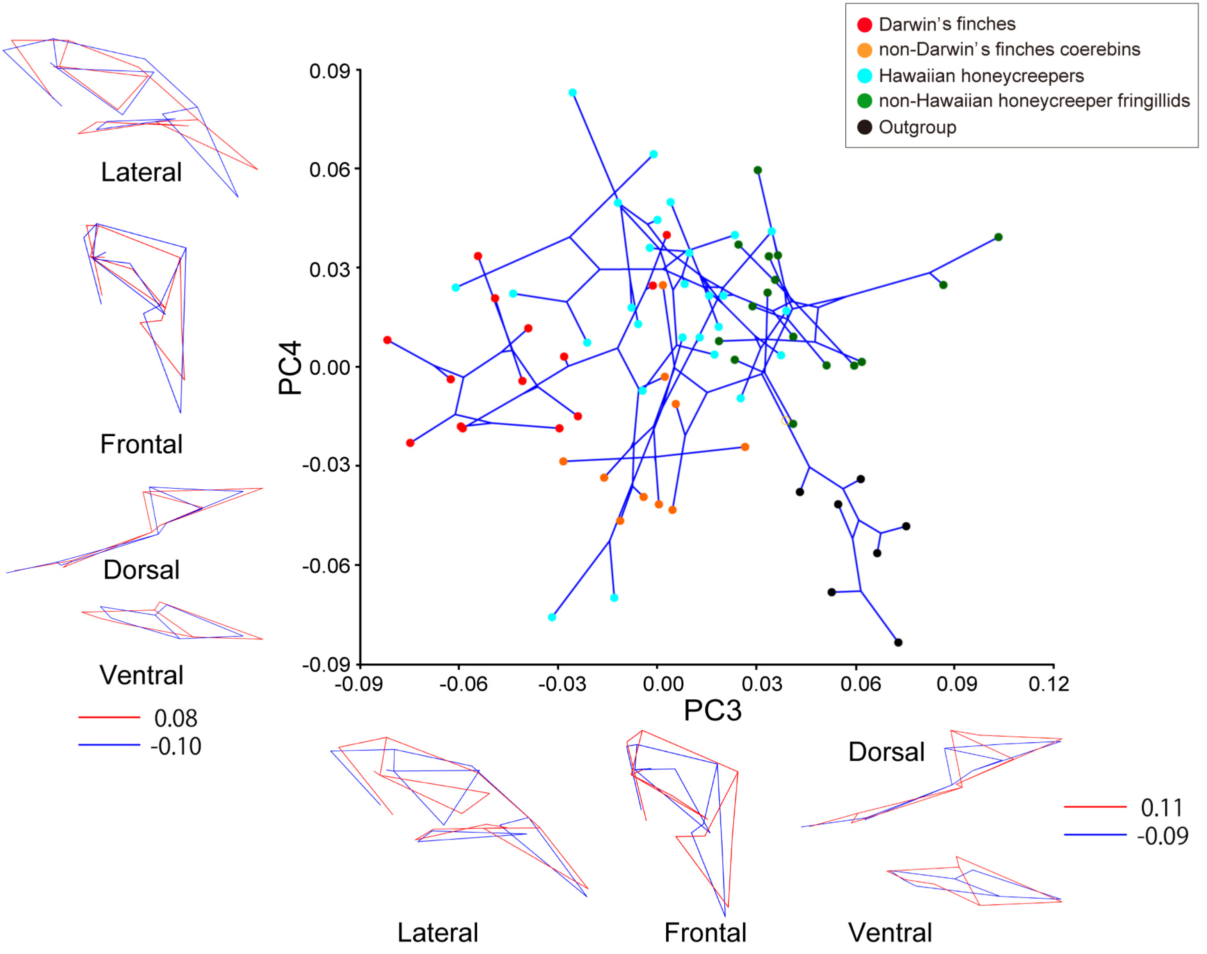

Supplementary Figure 4

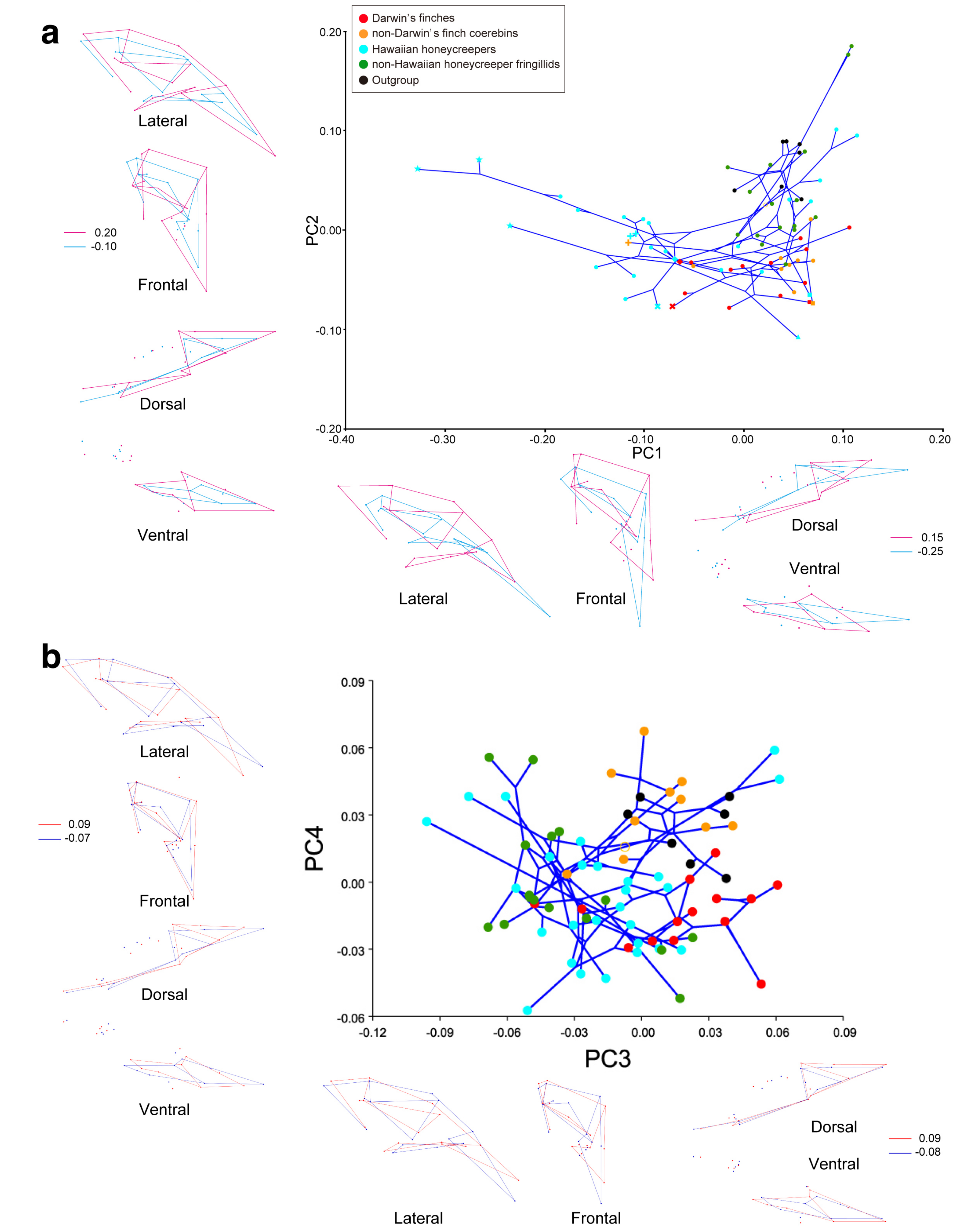

Supplementary Figure 5

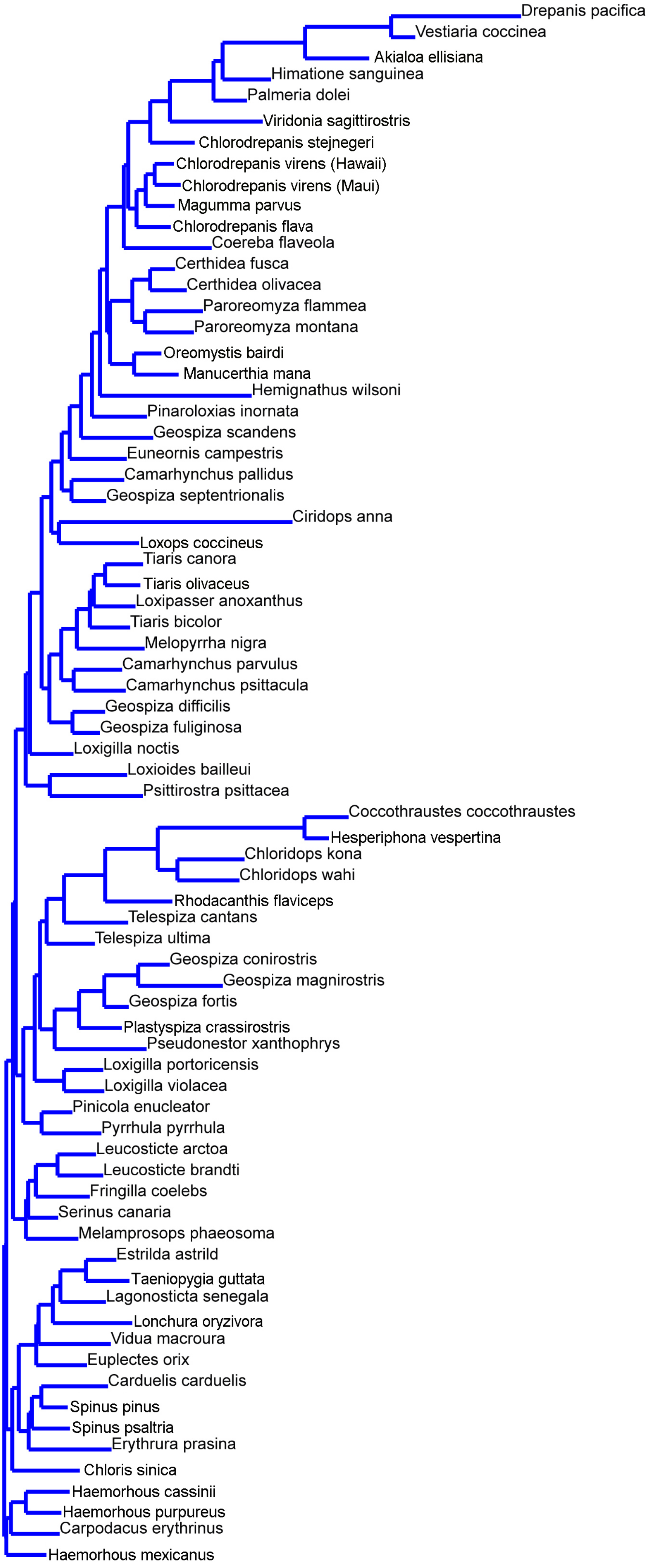

Supplementary Figure 6

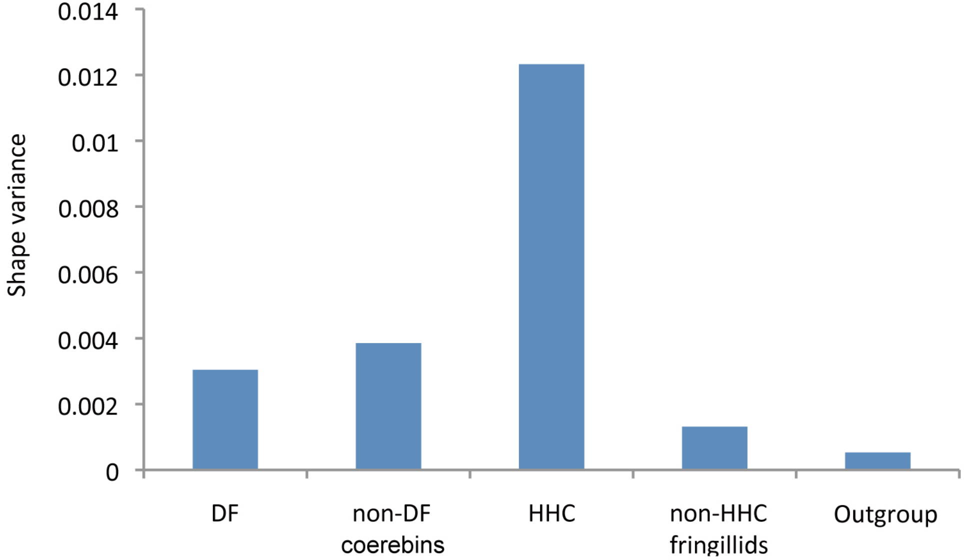

Supplementary Figure 7

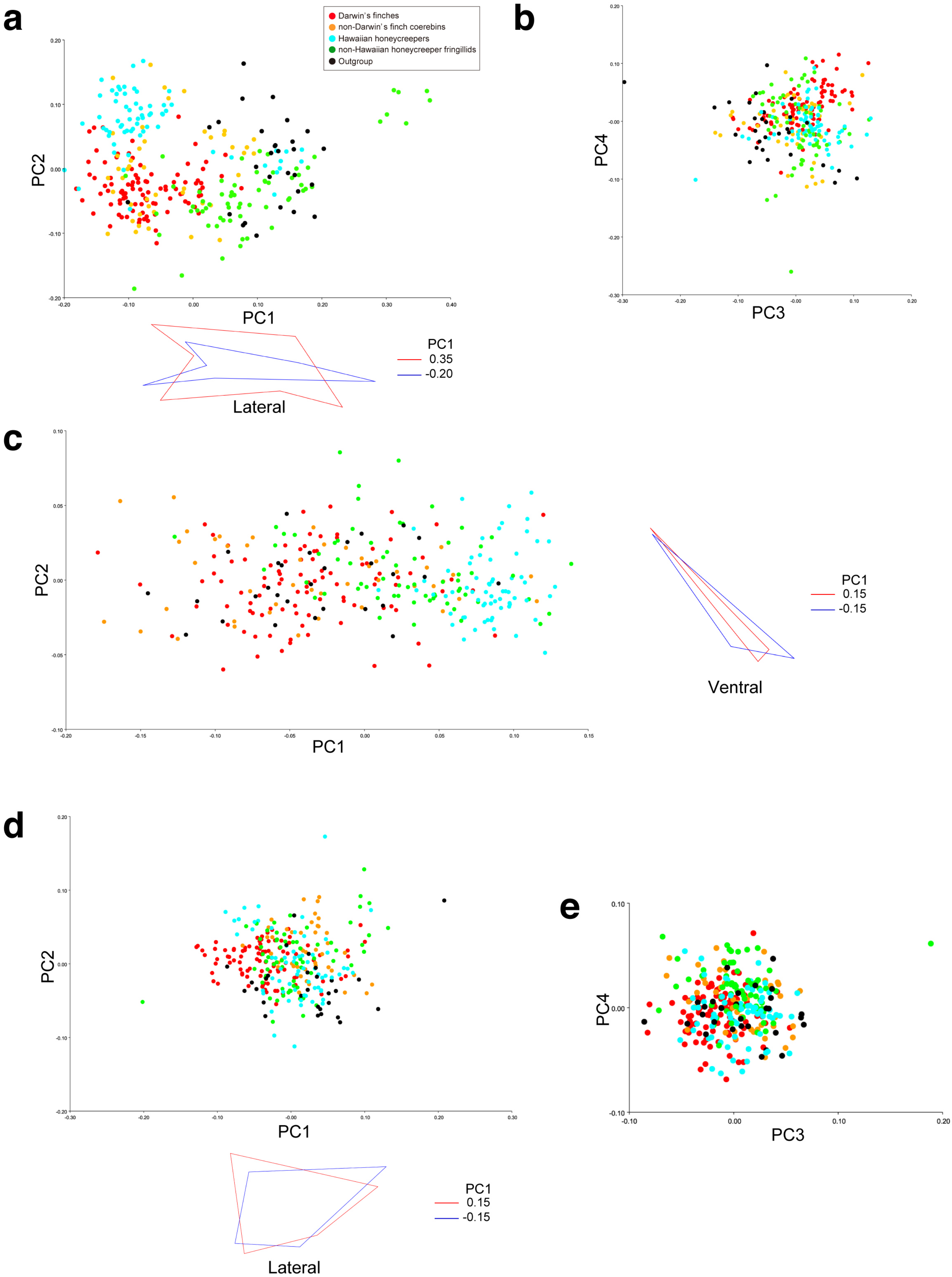

Supplementary Figure 8

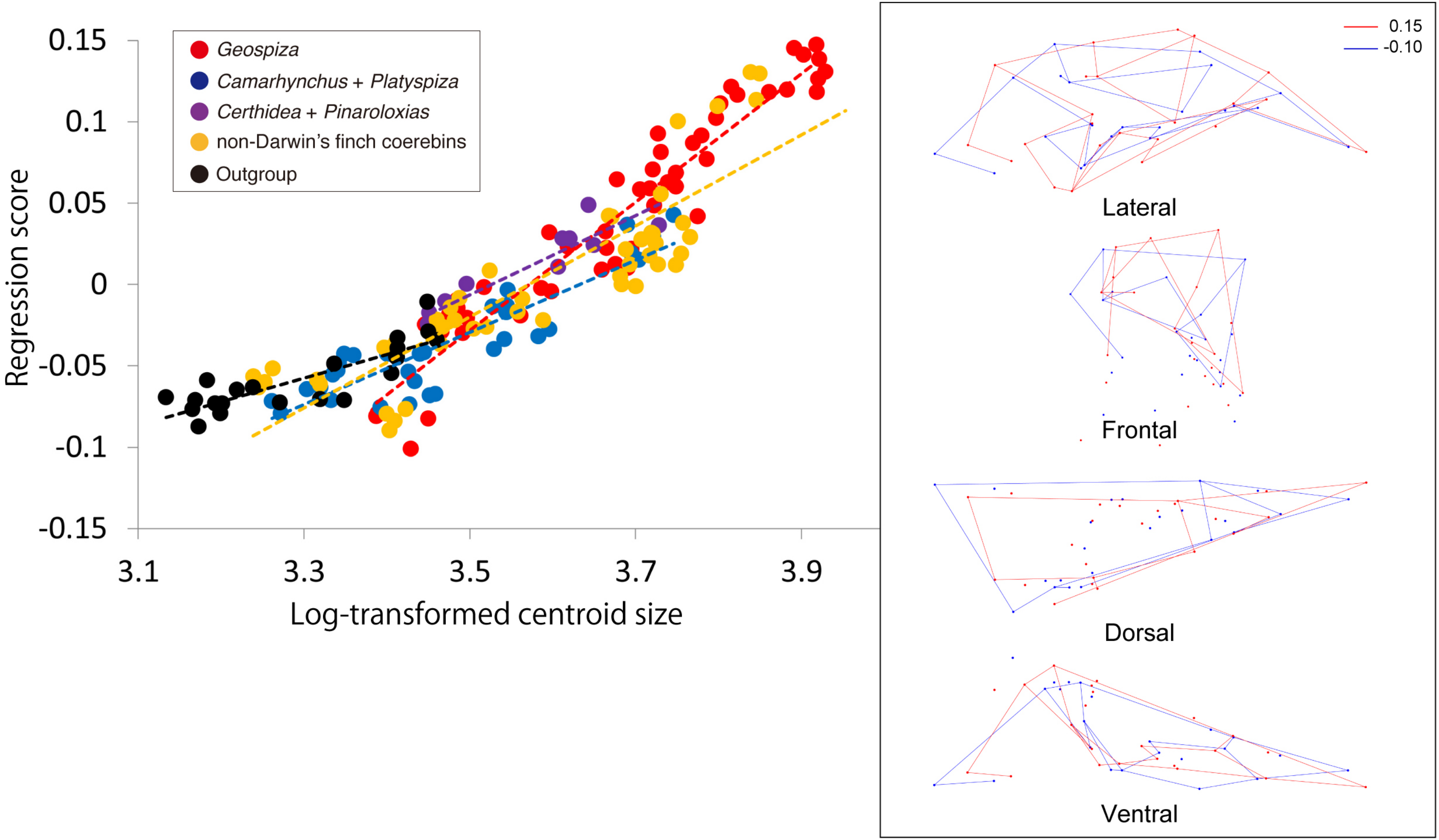

Supplementary Figure 9

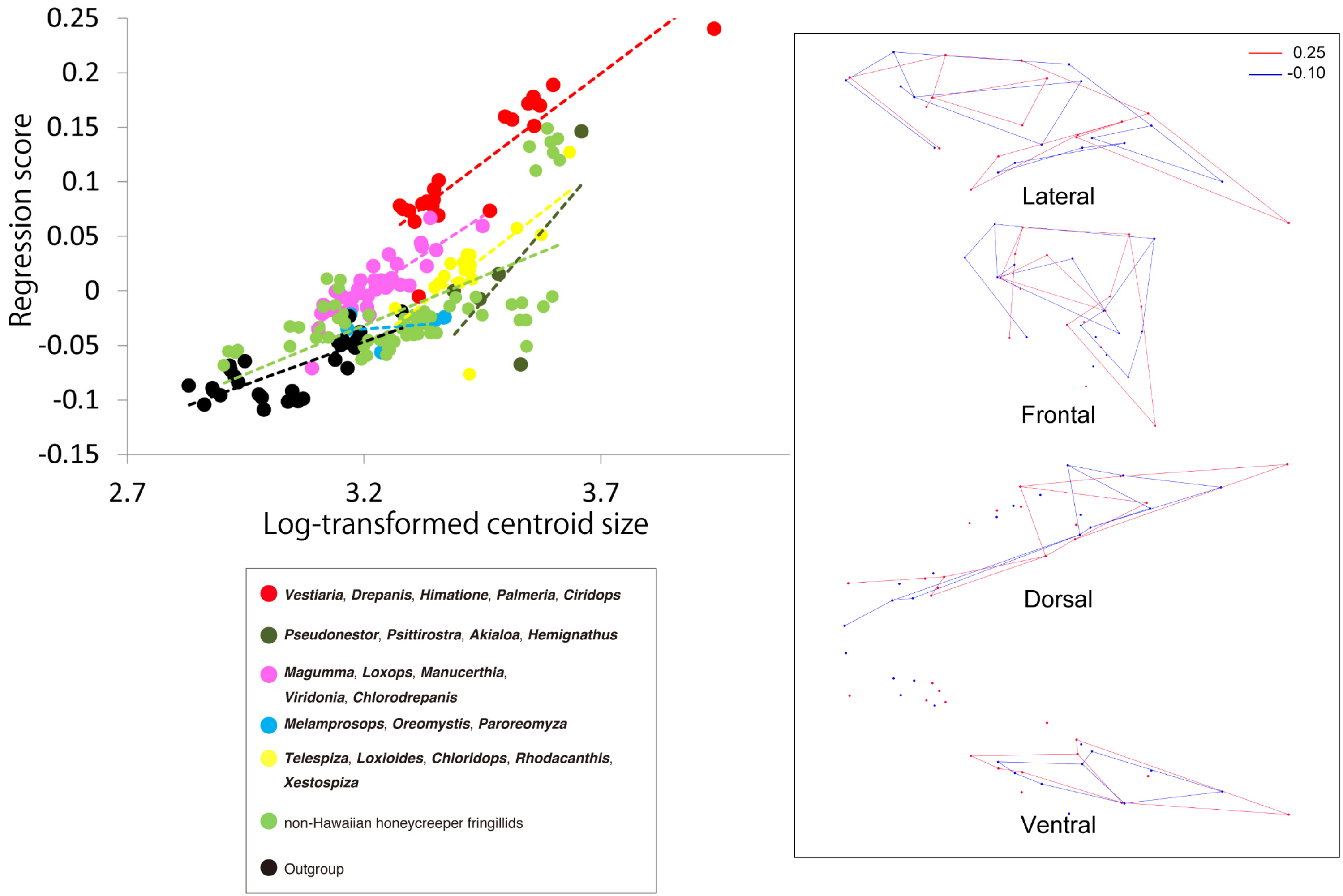

Supplement: Supplementary figures [file rstb20150481supp3.pdf]
